# Types of genotypes in progressive familial intrahepatic cholestasis and liver transplantation: A meta-analysis of observational studies

**DOI:** 10.1371/journal.pone.0350508

**Published:** 2026-06-01

**Authors:** Rania Sakka, Hela Abroug, Sabrine Ben Youssef, Mongi Mekki, Ridha M’rad

**Affiliations:** 1 Department of Genetics, Fattouma Bourguiba University Hospital, Monastir, Tunisia; 2 Research laboratory of Congenital Anomalies and Childhood Cancer LR12SP13, University of Monastir, Monastir, Tunisia; 3 Department of Preventive Medicine and Epidemiology, University of Monastir, Monastir, Tunisia; 4 Department of Pediatric Surgery, Fattouma Bourguiba University Hospital, Monastir, Tunisia; 5 Department of Congenital and Hereditary Diseases, Charles Nicolle University Hospital, Tunis, Tunisia; University Hospital of Bologna Sant’Orsola-Malpighi Polyclinic Department of Digestive System: Azienda Ospedaliero-Universitaria di Bologna Policlinico Sant’Orsola-Malpighi Dipartimento dell’apparato digerente, ITALY

## Abstract

**Background:**

Progressive familial intrahepatic cholestasis (PFIC) refers to a group of inherited cholestatic liver diseases that affect children, often leading to liver failure and requiring liver transplantation (LT). Many studies have established correlations between the effect of the causal gene variant types and the severity of the PFIC phenotype, the treatment considered, or its outcomes in patients. Nevertheless, no selection criteria for LT based on genotypes have been adopted for patients affected by this group of diseases. Therefore, we conducted a meta-analysis to investigate the association between the main PFIC subtype genotypes and the treatment with LT.

**Methods:**

Online databases were searched for articles on PFIC1–4 and LT. The Preferred Reporting Items for Systematic Reviews and Meta-Analyses guidelines were followed. The genotypes of patients were extracted from the included studies and categorized into a group of cases, harboring null genotypes, and a group of controls, harboring non-null genotypes. The relationship between the genotype type and LT outcome was expressed as an OR by assessing the LT event among the case group and the control group.

**Results:**

Eighteen studies involving 420 PFIC patients were included. A random-effects model was used to assess the OR. Overall, we observed a close relationship between the PFIC null genotype and the LT event; OR=2.79 (95% CI:1.63 to 4.77; p < 0.001). Subgroup analysis according to the PFIC subtype showed the same effect.

**Conclusions:**

Our results provide evidence of a potential association between null genotypes in PFIC diseases and the indication of LT as a treatment. Further trials are needed to confirm our results and guide decisions regarding personalized and early preventive LT.

**Research protocol registration:**

https://doi.org/10.17605/OSF.IO/MNQWB

## Introduction

Progressive familial intrahepatic cholestasis (PFIC) refers to a group of rare autosomal recessive diseases caused by pathogenic variants in genes that encode proteins controlling bile transport and expressed across hepatocyte membrane layers [1,2]. PFIC types 1, 2, 3, and 4 are the identified main types, due to mutations in *ATP8B1*, *ABCB11*, *ABCB4*, *TJP2*, respectively [1]. A variety of treatments exist for these conditions, which include both medical and surgical interventions [3]. Liver transplantation (LT) is considered when patients have failed medical treatment and/or biliary diversion or presented with refractory pruritus, end-stage liver disease, or carcinoma [3,4]. Many individual studies have observed correlations between PFIC genes’ biallelic truncating mutations and the severity of phenotype, the treatment considered, or its outcomes in patients. Indeed, tendencies for a better response to ursodeoxycholic acid (UDCA) or biliary diversion were observed among children carrying missense mutations of *ATP8B1*, *ABCB11*, or *ABCB4* genes compared to those with nonsense and truncating mutations [5–7]. In addition, recent studies from the multi-center North American research and from the NAtural course and Prognosis of PFIC and Effect of biliary Diversion (NAPPED) have categorized patients presenting with PFIC1 into groups of FIC1-A, FIC1-B, and FIC1-C, based on the number of predicted protein-truncating mutations, and it was found that patients with a FIC1-C genotype presented earlier and underwent surgical biliary diversion less frequently compared to those with FIC1-A and FIC1-B genotypes [8]. Moreover, patients with compound heterozygous or homozygous *ABCB11* mutations were stratified into BSEP1, BSEP2, and BSEP3 categories, based on the mildest predicted mutation, considering functional in vitro and genetic in silico data. Patients with BSEP1 mutations were found to have better outcomes and more frequent surgical biliary diversion than those with BSEP2 and BSEP3 mutations [9]. Interestingly, individuals with BSEP deficiency who carry one p.D482G or p.E297G mutation combined with one very severe protein-truncating mutation have a similar prognosis and disease course to that of patients with two severe mutations [10]. Despite the importance of the association between genotype and natural history, or outcomes, the selection criteria for LT candidates in PFIC diseases do not differ from those in patients with other liver diseases and no recommendations based on the individual genetic status have been established in the evaluation for LT [11]. In this study, we performed the first meta-analysis to systematically investigate the association between the PFIC genotype type and the need for LT.

## Methods

### Data sources and search strategy

The study was registered on the Open Science Framework platform (OSF; 10.17605/OSF.IO/MNQWB) and performed in accordance with the Preferred Reporting Items for Systematic Reviews and Meta-Analyses (PRISMA) guidelines [12] (S1 Table). We carried out a search on Medline (PubMed), Web of Science, Google Scholar, ScienceDirect, and Cochrane Library databases for articles published from inception up to 30 September 2024. The following PubMed syntax was used: (“Cholestasis, progressive familial intrahepatic 1” [Supplementary Concept]) OR “Cholestasis, progressive familial intrahepatic 2” [Supplementary Concept]) OR “Cholestasis, progressive familial intrahepatic 3” [Supplementary Concept]) AND (“ATP8B1 protein, human” [Supplementary Concept]) OR “ATP Binding Cassette Transporter, Subfamily B, Member 11”[Mesh]) OR “multidrug resistance protein 3” [Supplementary Concept]) OR “TJP2 protein, human” [Supplementary Concept]) OR “Mutation”[Mesh]) AND (“Liver Transplantation”[Mesh] OR “Hepatic transplantation”). Filters for Species (Human), Age (Child: birth-18 years), and Text availability (Free full text) were applied. Equivalent syntaxes were used for the remaining databases.

### Inclusion and exclusion criteria

The inclusion criteria were original observational studies, case series and cohort studies, either descriptive or analytical, and patients presenting with PFIC disease types 1–4 under the age of 18. The disease’s diagnosis was confirmed by molecular analysis. For studies reporting results of a common set of patients, only the most recent study with the largest sample size was selected.

Exclusion criteria were meta-analyses, case reports, reviews, conference abstracts, editorials, studies carried out on adult patients, and biological models. There were no language or date restrictions on the search.

### Study selection, data extraction and quality assessment

The author (R.S) performed the literature search and screened the records of titles and abstracts. Two authors (R.S) and (R.M) independently assessed the articles for eligibility for inclusion in the meta-analysis. The first reviewer (R.S) extracted study characteristics from the full text of the included articles. The second reviewer (R.M) checked the extracted data. Any detected discrepancies in selection and data extraction processes were resolved by consensus.

Data extracted included the type of PFIC, author, year of publication, country of the referral center, design of study, inclusion criteria, age of onset/diagnosis of the disease, disease’s presenting features, main diagnostic work-up, comorbidities, main indication(s) for LT, age at the time of LT, post-LT complications, age at last follow-up, genetic testing techniques, patients’ genotypes, variant pathogenicity assessment methods, variant nomenclature, number of LT carried out or indicated for the patients, treatments other than LT considered in patients and the binary outcome of the LT event in the groups of cases and controls. The quality of each included study was evaluated by the authors (R.S) and (H.A) independently and by using a checklist adapted from the National Heart, Lung, and Blood Institute (NHLBI) [10].

The NHLBI QAT for Observational Cohort and Cross-Sectional Studies and the NHLBI QAT of Case–Control Studies tools were used in accordance with the study design.

The studies were assessed in relation to bias due to selection of patients, measurement of exposure and outcomes, potential confounding and missing data due to loss to follow-up. The author (R.S) evaluated individual studies against the criteria in the NHLBI QAT tool and, this was cross-checked by the author (H.A).

### Genotype categorization

Extracted genotype data included variants according to gene reference sequence numbers: NM_005603 (ATP8B1); NM_003742 (ABCB11); NM_018849 or NM_000443 (ABCB4) and NM_004817 (TJP2). Variants were selected if they were predicted to be pathogenic or likely pathogenic and occurred in a biologically relevant transcript. Classification was based on two types: the null variants that included nonsense, frameshift, canonical splicing, or those located adjacent to the canonical splice sites, initiation codons, and single and multi-exon deletions. These variants can often be assumed to disrupt gene function by leading to the complete absence of the encoded protein, due to a lack of transcription or nonsense-mediated decay of an altered transcript [13–15]. The non-null variants included missense, synonymous and splicing variants, variants located at other splice sites, and non-frameshift small deletions. These variants are often partially functional, leading to decreased or reduced function of the protein [13,15].

The group of cases harbored null genotypes and the group of controls carried non-null genotypes. A null genotype included both alleles of the considered gene with null variant(s). A non-null genotype included at least one allele of the gene carrying only non-null variant(s).

### Statistical methods

The statistical analysis was conducted using the R software (version 4.3.1) [16] with the “meta” and “dmetar” packages to estimate the pooled frequency of the LT event among PFIC patients [17].

The relationship between the PFIC genotype type and the LT outcome was expressed as an OR by assessing the LT event among the null genotype cases group and the non-null genotype controls group. The “metafor” package in R was used to calculate the OR and the corresponding 95% CI, for individual studies as well as the overall pooled OR [17]. The results were plotted in forest plots. The level of statistical significance was set as p < 0.05.

Heterogeneity between studies was quantified using Cochran’s Q and the I^2^ tests. I^2^ values of 30% to 60%, 50% to 90% and 75% to 100% were interpreted as representing moderate, substantial, and considerable heterogeneity, respectively [18]. The random-effects model was used for the meta-analysis, in the case of significant heterogeneity across studies (p for Q test <0.1 and I^2^ > 50%). Forest plots summarized the effect estimates with 95% confidence intervals. Funnel plots, Egger’s test, Trim-and-fill test, and influence analysis by the leave-one-out method were used to detect publication bias [17,19].

In order to investigate confounding, the following sensitivity analyses were performed: (i) meta-regression using the Maximum Likelihood method [17] to explore the influence of the study design and HGVS nomenclature binary moderators; (ii) subgroup analysis stratifying articles by the covariates: study center/region, age at last follow-up ≤ or > 3 years as the median age at LT is 3.2 years [20]; UDCA use prior to LT and surgical treatment before LT; (iii) reclassification of the genotypes harboring the following borderline missense *ABCB11* variants: c.890A > G (E297G), c.1445A > G (D482G), c.2944G > A (G982R) and c.3628A > C (T1210P). These variants function similarly to null variants by causing protein degradation, trafficking defects, or loss of enzymatic activity [15]. Genotypes harboring these variants, in the homozygous state or in the compound heterozygous state with another null variant, were reclassified into null genotypes.

## Results

### Study selection

**Fig 1:**
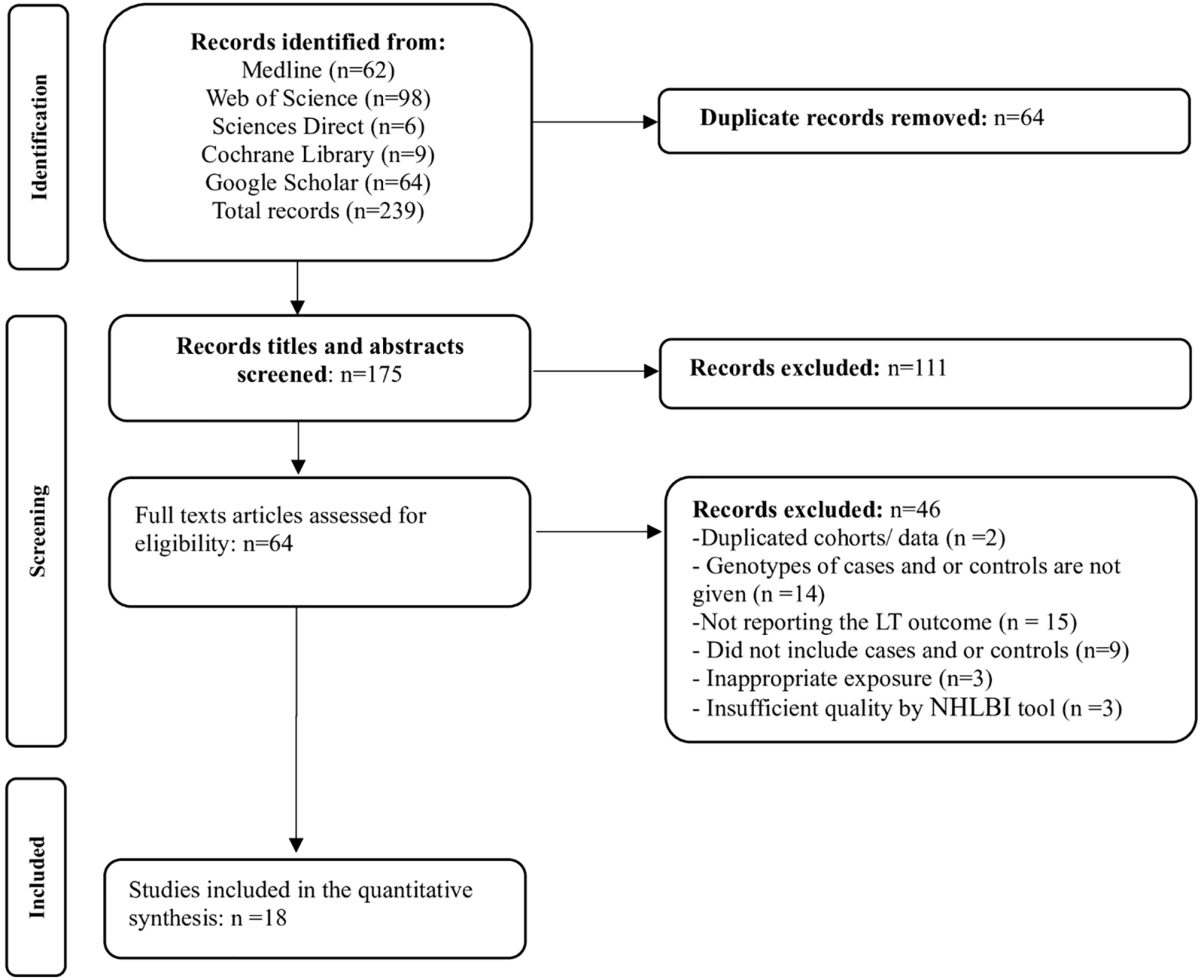
PRISMA-based flow diagram for meta-analysis.

The PRISMA flow diagram for selecting studies is summarized in Fig 1. In total, 239 records were identified through database searches. After removing all duplicates (64), we screened the titles and abstracts of 175 articles, of which 64 articles were further reviewed in full-texts. Of these, 18 articles [20–35] met the inclusion criteria and were selected for the meta-analysis.

### Study characteristics

Table 1 shows the main characteristics of the included studies. They were published between 2000 and 2023; 13 were cohort studies and 5 were case series. Nine studies were conducted in Europe [20,21,25,27,28,30,33,35]; 5 in Asia [29,32,34,36,37]; 2 in USA and UK [23,24]; 1 in the USA, UK, and Canada [26]; and 1 was multicentric, representing data from 12 countries across Europe and Asia [31].

**Table 1:** Characteristics of the included studies.

Author (year)	Country of the referral center(s)	Study design	Genetic testing techniques	Variant pathogenicity assessment methods	Variant nomencl-ature	Medical treatment prior to LT	Invasive surgery prior to LT	Age at indication of LT	n of all genotyped patients	n of patients (LT+)	n cases with null genotype		n controls with non-null genotype	
											**(LT+)**	**(LT-)**	**(LT+)**	**(LT-)**
**Klomp et al.2000 [21]**	Greenland	Case series	SSCPA, DNA Sanger sequencing and RFLPA	Genotyping and assessment of variant frequency in normal controls	Other than HGVS	NR for all patients	NR	6 y	PFIC1: 13	PFIC1: 1	PFIC1: 0	PFIC1: 0	PFIC1:1	PFIC1: 12
**Chen et al. 2002 [36]**	Taiwan	Prospecti-ve cohort	DNA Sanger sequencing	Genotyping and assessment of variant frequency in normal controls	Other than HGVS	NR for all patients	NR	5.5 y	PFIC1: 3 PFIC2: 2	PFIC1: 1 PFIC2: 0	PFIC1: 1 PFIC2: 0	PFIC1: 0 PFIC2: 0	PFIC1: 0 PFIC2: 0	PFIC1: 2 PFIC2: 2
**Knisely et al. 2006 [22]**	UK	Retrospec-tive cohort	Direct DNA Sanger sequencing	Liver immunohistochemistry, haplotype analysis for patient and donor	Other than HGVS	NR for all patients	1 case: hepatocyte infusion 3 cases: PEBD 5 cases: None	6 cases: ages between 13 mo and 29 mo	PFIC2:9	PFIC2:6	PFIC2:3	PFIC2:1	PFIC2:3	PFIC2:2
**Strautnieks et al. 2008 [23]**	UK,USA	Retrospec-tive cohort	SSCPA, Sanger sequencing and RFLPA	Liver immunohisto-chemistry, genotyping, family haplotype analysis and assessment of variant frequency in normal controls	HGVS	UDCA reported for 7 patients	41 cases: PEBD/ileal exclusion	53 cases: ages between 0.9 y and 19 y (median: 3.45 y)	PFIC2: 120	PFIC2: 53	PFIC2: 13	PFIC2:9	PFIC2:40	PFIC2: 58
**Evason et al. 2011 [24]**	USA, UK	Retrospec-tive cohort	Direct DNA Sanger sequencing	Liver immunohistochemistry	HGVS	NR for all patients	5 cases: Biliary diversion/ileal exclusion	7 cases: ages between 17mo and 88mo	PFIC2:11	PFIC2:7	PFIC2:3	PFIC2:0	PFIC2:4	PFIC2:4
**Colombo et al. 2011 [25]**	Italy	Retrospec-tive cohort	DNA Sanger sequencing and multiplex ligation–dependent probe amplification	Liver immunohisto-chemistry, Genotyping and assessment of variant frequency in normal controls and in silico analysis	HGVS	UDCA for all patients	None	5 cases: ages between 6 y and 17 y (median 6 y and 10 mo)	PFIC3:20	PFIC3:5	PFIC3:1	PFIC3:1	PFIC3: 4	PFIC3:14
**Sambrotta et al.2014 [26]**	UK, USA, Canada	Retrospec-tive cohort	NGS and DNA Sanger sequencing	mRNA expression, western blot and immunohistochemistry	HGVS	NR for all patients	PEBD for 1 patient (age NR)	9 cases: ages between 1.5 y and 10 y (median at 4 y)	PFIC4:12	PFIC4:9	PFIC4: 9	PFIC4:3	PFIC4:0	PFIC4:0
**Giovannoni et al. 2015 [27]**	Italy	Retrospec-tive cohort	DNA sanger sequencing and qPCR	In silico analysis	HGVS	UDCA for all patients	biliary diversion for 1 patient (age NR)	NR	PFIC1: 5 PFIC2: 17 PFIC3: 3	PFIC1: 5 PFIC2: 8 PFIC3:2	PFIC1: 4 PFIC2:1 PFIC3: 2	PFIC1: 0 PFIC2: 2 PFIC3: 0	PFIC1: 1 PFIC2: 7 PFIC3: 0	PFIC1: 0 PFIC2: 7 PFIC3: 1
**Schatz et al.2018 [28]**	Germany	Retrospec-tive cohort	DNA Sanger sequencing	In silico analysis	HGVS	UDCA reported for 23 patients	None	15 cases: ages between 3 mo and 16y (median at 6.6 y)	PFIC3:25	PFIC3:16	PFIC3: 3	PFIC3:0	PFIC3:13	PFIC3:9
**Kang et al. 2019 [37]**	Korea	Case series	Direct Sanger DNA sequencing	In silico analysis	HGVS	UDCA reported for one patient	NR	NR	PFIC1: 1 PFIC2: 2	PFIC1: 1 PFIC2: 1	PFIC1: 0 PFIC2: 1	PFIC1: 0 PFIC2: 0	PFIC1: 1 PFIC2: 0	PFIC1: 0 PFIC2: 1
**Zhang et al. 2020 [29]**	China	Case series	Direct Sanger DNA sequencing	In silico analysis	HGVS	NR for all patients	NR	2 cases: 1) 2 y and 8 mo 2)4 y and 5 mo	PFIC1: 1 PFIC2: 4 PFIC3: 4	PFIC1: 1 PFIC2: 1 PFIC3: 0	PFIC1: 0 PFIC2: 0 PFIC3: 0	PFIC1: 0 PFIC2: 1 PFIC3: 1	PFIC1: 1 PFIC2: 1 PFIC3: 0	PFIC1: 0 PFIC2:2 PFIC3: 3
**Lipi´nski et al.2020 [30]**	Poland	Historico- prospecti-ve cohort	NGS	assessment of variant frequency in normal population and in silico analysis	HGVS	UDCA for all patients	PEBD for 4 patients	3 cases: 1)1 y 4mo 2)3 y 6 mo 3)12 y	PFIC2:8 PFIC3: 2 PFIC4: 2	PFIC2:2 PFIC3: 1 PFIC4: 0	PFIC2:0 PFIC3: 0 PFIC4: 0	PFIC2:1 PFIC3: 0 PFIC4: 1	PFIC2:2 PFIC3: 1 PFIC4: 0	PFIC2:5 PFIC3: 1 PFIC4: 1
**Jeyaraj et al.2021 [31]**	multi-center*	Prospecti-ve cohort	Microarray resequencing and NGS	In silico analysis	HGVS	NR for all patients	PEBD for 4 patients	NR	PFIC1: 3 PFIC2: 10 PFIC3: 4	PFIC1: 1 PFIC2: 3 PFIC3: 1	PFIC1: 1 PFIC2: 0 PFIC3: 0	PFIC1: 0 PFIC2: 2 PFIC3: 1	PFIC1: 0 PFIC2: 3 PFIC3: 1	PFIC1: 2 PFIC2:5 PFIC3: 2
**Al-Hussaini et al. 2021 [32]**	Saudi Arabia	Retrospec-tive cohort	Targeted NGS or whole-exome sequencing	In silico analysis	HGVS	UDCA for all patients	NR	25 cases: ages between 0.83 y and 11 y (median 3.5 y)	PFIC1: 3 PFIC2: 31 PFIC3: 25	PFIC1: 2 PFIC2: 16 PFIC3: 7	PFIC1: 2 PFIC2: 8 PFIC3: 3	PFIC1: 0 PFIC2: 8 PFIC3: 0	PFIC1: 0 PFIC2: 8 PFIC3: 4	PFIC1: 1 PFIC2: 7 PFIC3: 18
**Lipi´nski et al.2021 [38]**	Poland	Case series	NGS	In silico analysis	HGVS	NR for all patients	NR	2 cases: 1) 18 y 2) 12 y	PFIC3:4	PFIC3:2	PFIC3:0	PFIC3:0	PFIC3: 2	PFIC3:2
**Chen et al. 2022 [34]**	China	Case series	NGS	In silico analysis	HGVS	UDCA for all patients	NR	NR	PFIC3:5	PFIC3:1	PFIC3:0	PFIC3:2	PFIC3:1	PFIC3:2
**Pfister et al. 2023 [20]**	Germany	Retrospec-tive cohort	Targeted NGS or whole-exome sequencing	In silico analysis	HGVS	Reported for 3 cases: nasobiliary tube or molecular adsorbent recirculating system therapy	Biliary diversion surgery for 18 patients	22 cases: ages between 5 mo and 23.8 y (median 3.2 y)	PFIC2: 46	PFIC2: 22	PFIC2: 6	PFIC2: 0	PFIC2: 16	PFIC2:24
**Sahloul et al. 2023 [35]**	Germany	Retrospec-tive cohort	NR	In silico analysis	Other than HGVS	UDCA for all patients	PEBD/ mean age 4.31 (± 4.92)	19 cases: mean age 7.08 y (± 5.92)	PFIC1: 3 PFIC2: 16 PFIC3: 6	PFIC1: 2 PFIC2:12 PFIC3: 5	PFIC1: 2 PFIC2: 3 PFIC3: 0	PFIC1: 0 PFIC2: 0 PFIC3: 0	PFIC1: 0 PFIC2:9 PFIC3: 5	PFIC1: 1 PFIC2: 4 PFIC3: 1

Multi-center*. countries of Bulgaria, Canada, Denmark, Germany, Greece, Hungary, India, Netherlands, Oman, Poland, Turkey and the UK.

Abbreviations: HGVS: Human Genome Variation Society; LT: liver transplantation; LT + : underwent liver transplantation; LT-: not underwent liver transplantation; mo: months; NGS: Next-generation sequencing;NR: not reported; n: number; PEBD: Partial external biliary diversion; PFIC1: Progressive familial intrahepatic cholestasis type 1; PFIC2: Progressive familial intrahepatic cholestasis type 2; PFIC3: Progressive familial intrahepatic cholestasis type 3; PFIC4: Progressive familial intrahepatic cholestasis type 4; RFLPA: Restriction Fragment Length Polymorphism; SSCPA:Single-Strand Conformation Polymorphism Analysis; y: years; UDCA: Ursodeoxycholic acid.

The included studies reported either next-generation, Sanger sequencing, or a combination of the two as the genetic sequencing technique. One study did not report that information [35].

A total of 420 PFIC patients were involved across all the studies. There was considerable variability in studies regarding the sample size (from 3 to 120). All patients included were homozygotes or compound heterozygotes for variants of PFIC genes predicted to be pathogenic or likely pathogenic (S3 Table). All studies reported gene variants according to the Human Genome Variation Society (HGVS) or older mutation nomenclature recommendations.

For the ascertainment of the variants’ pathogenicity, ten studies used only computational evidence [20,27–29,31–35,37], whereas eight assessed the frequency of a variant in a control or in the general population in combination with mRNA and protein expression technical analysis approaches [21–26,30,36].

### Assessment of risk of bias

When evaluated by the NHLBI quality assessment tool [39], most studies were rated as being fair (n = 10) or good (n = 8) in quality (S2 Table). Three studies [5,7,40] were rated as of poor quality and excluded mainly because they did not display the outcome (LT event) and the exposure (genotype status) for each patient.

As shown in Table 1 and S4 Table, all studies reported children with PFIC who underwent LT (LT+) from the whole sample of patients initially recruited. The most important sources of bias were in the classification of the outcomes due to losses to follow-up or the limited study duration necessary to observe outcomes, especially among patients recruited at the younger ages. Potential confounding factors related to both the pharmacological treatment and interventions before considering or carrying out LT in patients were fully reported only in 7 studies and consisted mainly of UDCA and biliary diversion without details about its duration [20,23,25,27,28,30,35]. Responses to UDCA ranging from complete, partial, ineffective or negative were detailed for all patients from the same sample only in 4 studies involving a total of 112 patients [25,28,30,32]. Also, nutritional status, regarding growth failure and or malabsorption, was only reported for 47 patients, from 10 studies [21,22,24,25,27–29,32,34,36]. None of the studies reported comorbidity scores (e.g., PELD/MELD). Concurrent diseases with PFIC, including inborn errors of metabolism, infectious diseases, neonatal hepatitis and ductal malformations, were excluded in 9 studies [20–22,24,25,27,34]. None of the included studies stated ruling out autoimmune liver disease concurrent with PFIC by performing immunological tests. However, available liver histological features from 11 studies [20–25,27,29,30,33,34,36] did not include histologic signs described in autoimmune hepatitis [41–43]. The indication of LT was reported for 76 patients from 11 studies and consisted of end stage liver disease [21,38]; severe cirrhosis [24,35,36,38]; cholestasis [22]; biliary diversion failure [22]; persistent pruritus [20,24]; portal hypertension, coagulopathy and ascites [24]; liver failure [34]; severe liver failure and pruritus [28]; portal hypertension and severe pruritus; severe cholestasis, portal hypertension and liver failure; severe cholestasis and portal hypertension. The post-LT complications were reported for 14 patients from six studies and were: intractable diarrhea [21]; mild diarrhea [36]; death from sepsis or liver failure [22,28]; graft rejection [28,37]; biliary complications [28]; steatohepatitis and recurrence of PFIC2 [37].

## Data synthesis and Meta‑analysis

### Estimating the frequency of LT outcome among PFIC patients

**Fig 2:**
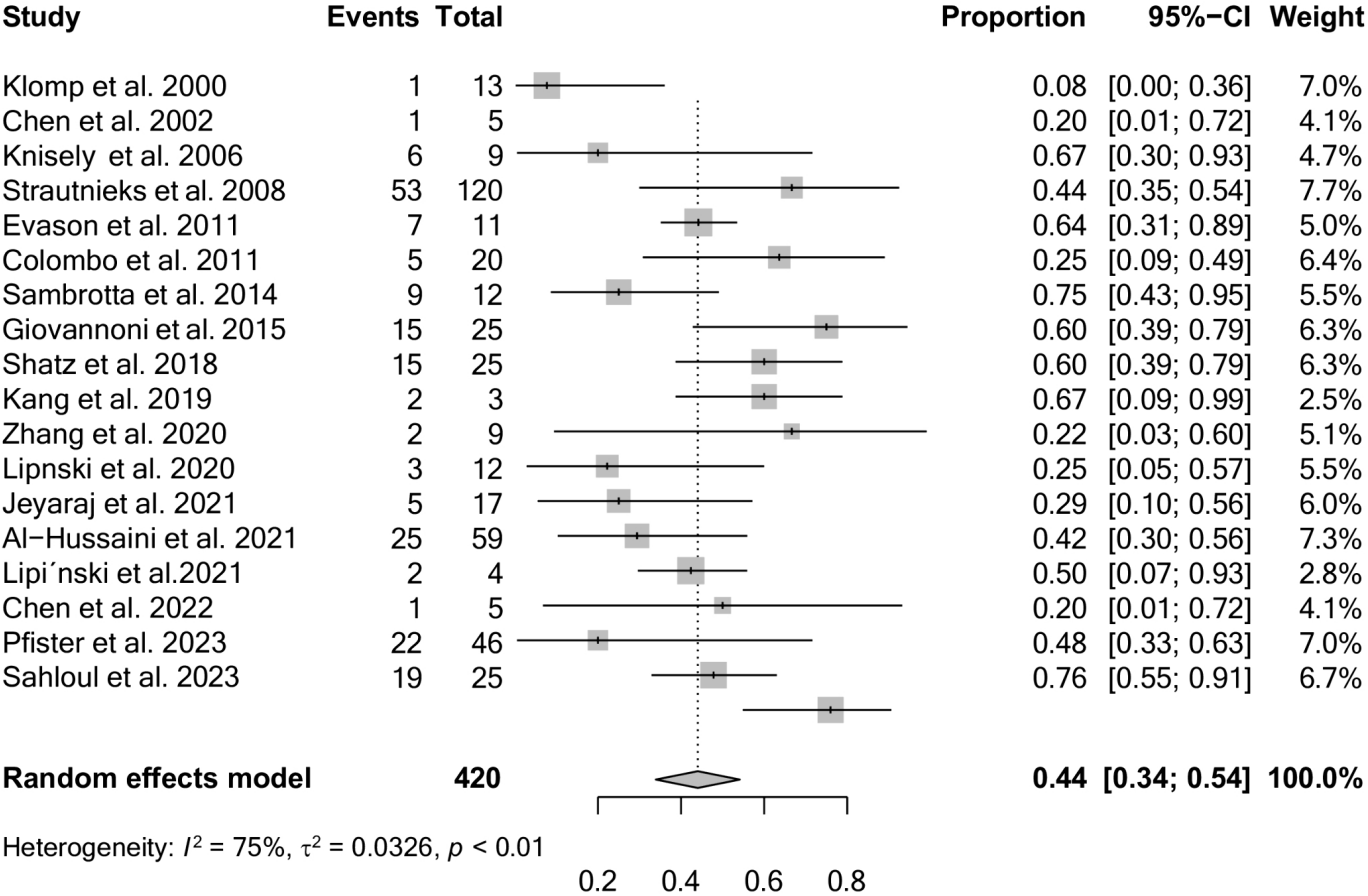
Forest plot of the overall pooled frequency of LT outcome.

In total, 193 patients (LT+) were reported among 420 patients presenting with PFIC disease. The overall pooled prevalence of all patients (LT+) amongst all PFIC patients was 44% (95% CI: 34% to 54%) (Fig 2).

**Fig 3:**
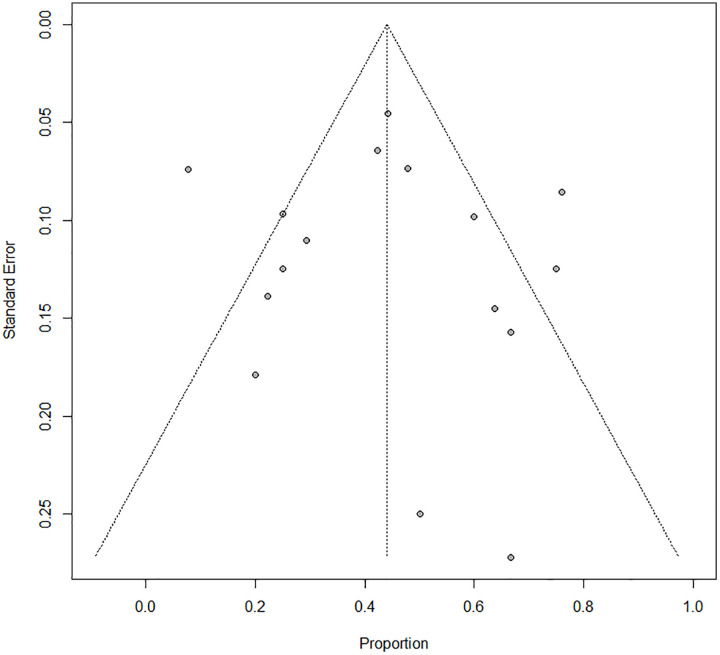
Funnel plot of the overall pooled frequency of LT outcome.

Considerable heterogeneity was observed between the 18 studies (I^2^ = 75%; p < 0.001). The Egger’s test did not suggest the presence of publication bias (intercept: 0.272; 95% CI: −1.99 to – 2.53; t: 0.236; p: 0.82). The funnel plot is shown in Fig 3.

**Fig 4:**
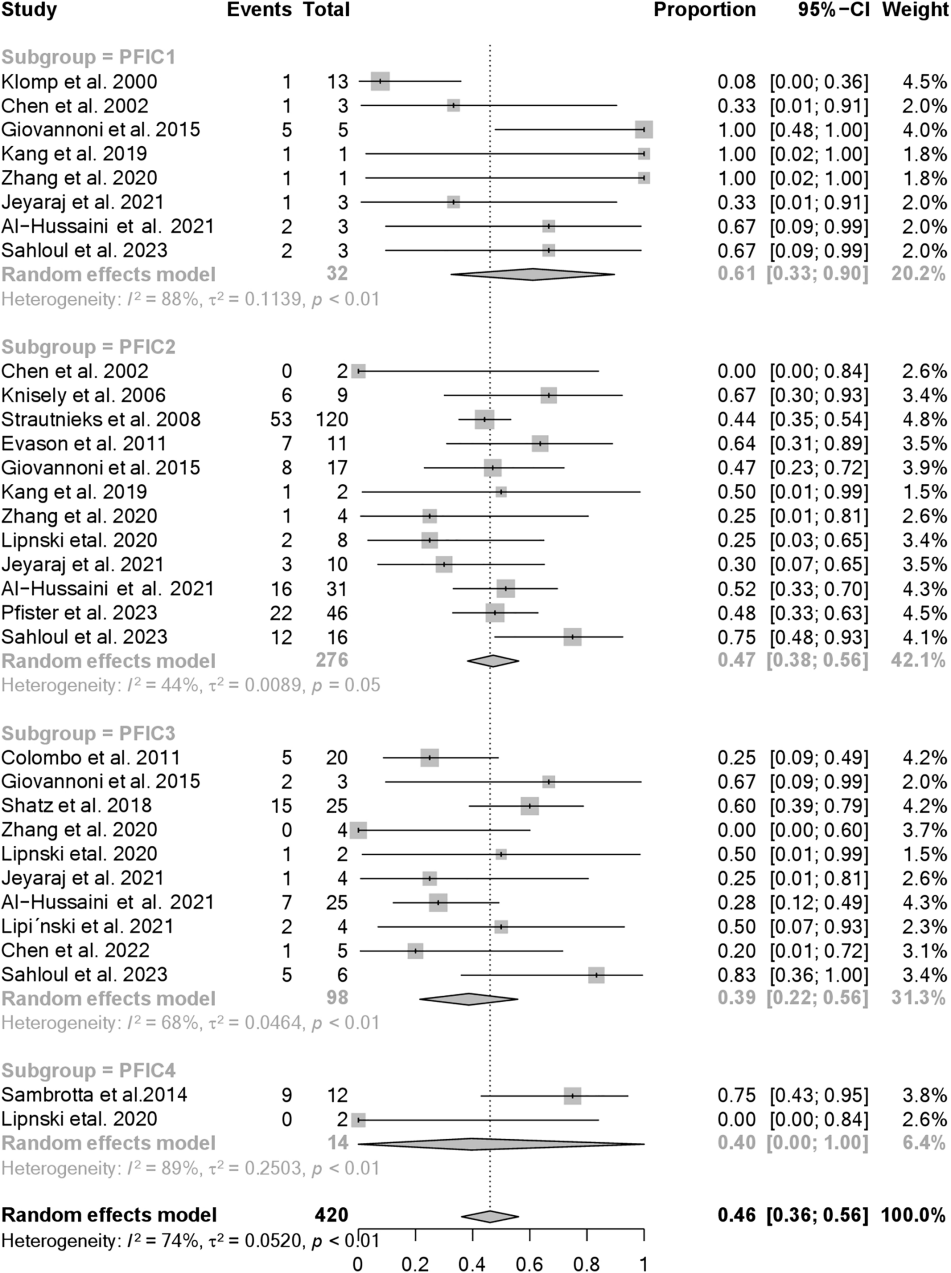
The pooled frequency of patients (LT+) stratified by PFIC subtype.

The pooled proportions of patients (LT+) stratified by PFIC type were 61% (95% CI: 33% to 90%, I^2^: 88%) for PFIC1, 47% (95% CI: 38% to 56%, I^2^: 44%) for PFIC2, 39% (95% CI 22% to 56%, I^2^: 59%) for PFIC3 and 40% (95% CI: 0% to 100%, I^2^: 89%) for PFIC4 (Fig 4).

### Evaluating the relationship between the genotype type and the LT outcome

The studies included 105 patients in the cases group (null genotype) and 315 patients in the controls group (non-null genotype). To account for the variability between studies regarding the frequency of LT outcome, a random-effects model was used to assess the overall OR.

**Fig 5:**
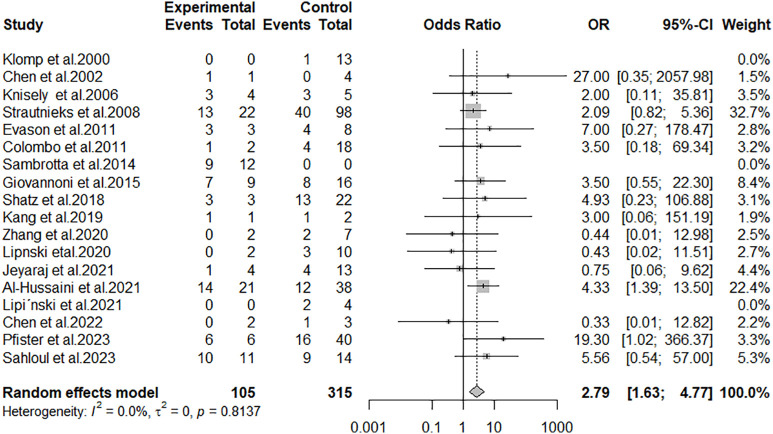
Forest plot of the overall pooled OR of LT outcome.

The value of the overall pooled OR=2.79 (95% CI: 1.63 to 4.77; p < 0.001) indicated a higher frequency of (LT+) outcome in the case group than in the control group and suggested a significant association between the PFIC null genotype and the (LT+) outcome (Fig 5).

**Fig 6:**
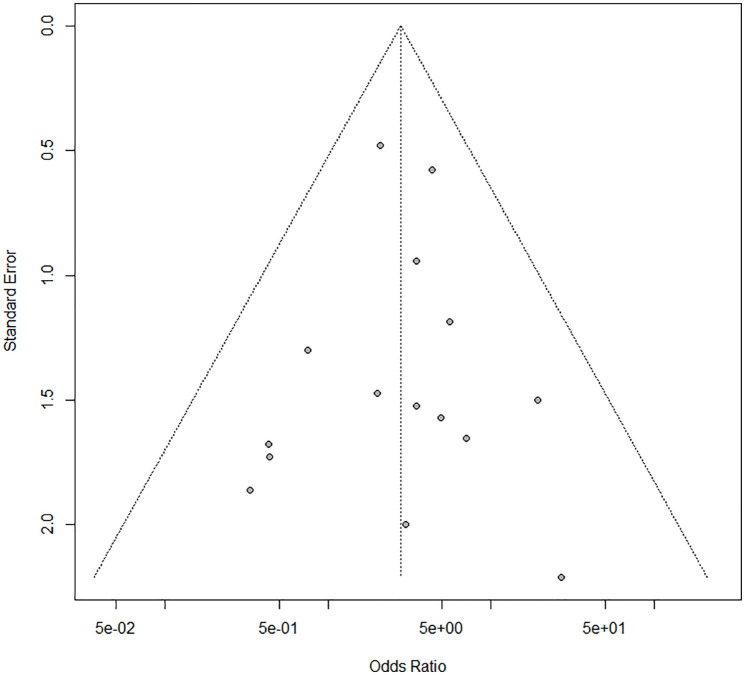
Funnel plot of the overall pooled OR of LT outcome.

Heterogeneity between studies might not be important (I^2^ = 0%; p = 0.81). The funnel plot (Fig 6) and Egger’s test did not suggest the presence of publication bias (intercept: – 0.043; 95% CI: −0.93 to – 0.85; t: – 0.095; p: 0.92).

The trim-and-fill analysis revealed that no studies needed to be trimmed or filled to adjust the overall effect size. The leave-one-out method showed that no study influenced the overall OR (S5 Fig.).

To investigate how the genotype’s effect varied across PFIC types, we performed a subgroup analysis.

**Fig 7:**
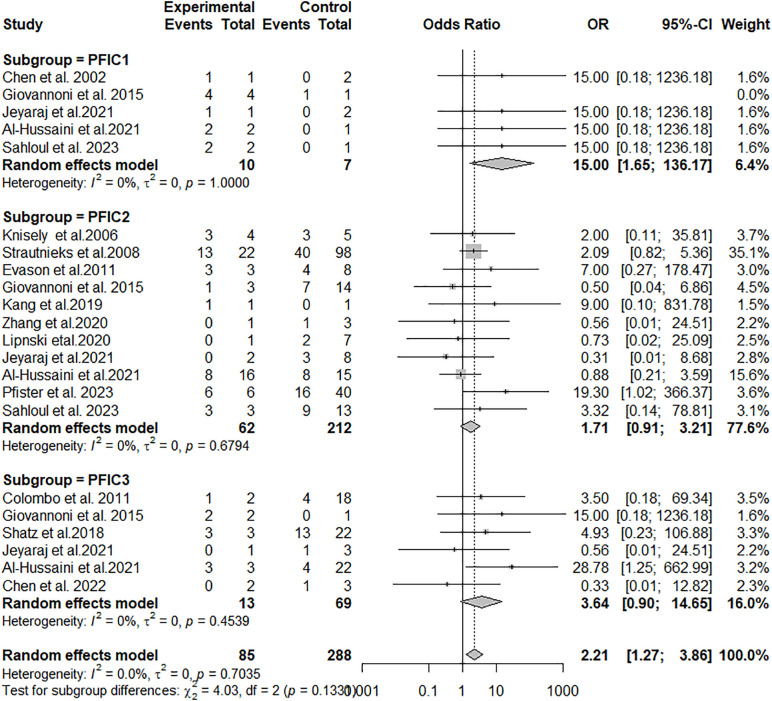
The pooled frequency of OR stratified by PFIC subtype.

In each subgroup, we omitted studies with non-positive values for the cases group and/or for the controls group because they received no weight in the meta-analysis, and we excluded the PFIC4 subgroup because there was only one study involving PFIC4 disease. The results of the subgroup analyses for PFIC1, PFIC2 and PFIC3 are presented in Fig 7.

No statistically significant quantitative subgroup effect was noted (p = 0.13), suggesting that the PFIC subtype does not modify the effect in the null genotype group in comparison with the non-null genotype group. However, a smaller number of studies and participants contributed to PFIC1 and PFIC3 subgroups than to the PFIC2 subgroup, meaning that the analysis may not be able to detect subgroup differences.

The quality of the evidence was rated as low because all studies included in the quantitative synthesis had an observational design.

### Sensitivity analysis

The meta-regression indicated that neither study design nor use of HGVS nomenclature significantly moderated the effect size (overall test of moderators p > 0.05). Individually, study design (p = 0.33) and HGVS nomenclature (p = 0.44) showed no significant impact on the pooled effect.

Subgroup analyses for age at last follow-up and surgical intervention before LT showed no statistically significant subgroup differences (p = 0.35 and p = 0.19, respectively) (S6 Fig and S7 Fig). The effect of LT consistently favored the null genotype over the non-null genotype across all subgroups. However, these subgroup analyses were likely underpowered, as substantially more participants were included in the age > 3 years subgroup (n = 180) than in the age ≤ 3 years subgroup (n = 90), and in the no-surgery-before-LT subgroup (n = 236) than in the surgery-before-LT subgroup (n = 86). Therefore, the analyses may not have been able to detect differences in effect size between subgroups.

Subgroup analysis for the covariate “UDCA use prior to LT” was not considered valid, as only four participants contributed to the no-UDCA-prior to LT subgroup (data not shown). The very small sample size resulted in inadequate statistical power to detect any meaningful subgroup differences.

Subgroup analysis by study center/region showed no statistically significant differences between subgroups (p = 0.64) and no evidence of between-subgroup heterogeneity (S8 Fig). The LT effect consistently favored the null genotype over the non-null genotype across all subgroups. However, the Asia subgroup included fewer participants (n = 81) than the Europe (n = 179) and multicenter (n = 160) subgroups. Consequently, the subgroup analyses may have been underpowered to detect true differences in effect size between regions.

Following the reclassification of 53 borderline non-null genotypes into null genotypes, the overall OR measure was 2.69 (95% CI: 1.70 to 4.25; p < 0.001) (S9 Fig), near similar to the initial overall OR of 2.79 (95% CI: 1.63 to 4.77; p < 0.001). This was consistent with a stable underlying type of genotype–LT effect across studies.

## Discussion

We performed this meta-analysis of 18 observational studies to assess the relationship between the genotype and indication for LT across the main PFIC subtypes. To the best of our knowledge, this is the first meta-analysis exploring such an association. We first found that (LT+) was a frequent event among all PFIC patients, mainly in the PFIC1 subtype. The same finding was reported in a previous systematic review [44], thus making statistical error modelling less likely. Also, PFIC2 was the largest subgroup among patients included in the meta-analysis. This predominance was also noted in a recent systematic review of the epidemiology, natural history and burden of PFIC [45].

Second, quantitative analysis showed that PFIC patients carrying null genotypes are associated with significantly increased odds of LT intervention, meaning that patients with this type of genotype were more likely to undergo LT. Moreover, subgroup analyses according to the PFIC type did not demonstrate differences. The narrow CI of the PFIC2 subgroup effect increases our confidence in the global effect estimate despite the fewer studies included in the PFIC1 and PFIC3 subgroups and the wide CI found for both of them.

Our findings are in line with those of NAPPED studies, which provide evidence of the highest frequency of LT among PFIC2 patients harboring biallelic truncating mutations [40] and lower native liver survival among PFIC2 patients harboring null genotypes compared with those harboring non-null genotypes [9,10].

Also, earlier genotype-phenotype correlation studies corroborate our results as they found that LT was necessary for all PFIC3 patients with a deletion or stop codon and in half of those with a missense mutation [6,7].

Existing evidence has suggested that PFIC2–4 genes cause disease through a protein loss-of-function mechanism [26]. Protein-truncating mutations in *ABCB11* and *ABCB4* result in the poorest membrane expression of the respective canalicular bile acid transport proteins BSEP and MDR3 [35,36]. Protein-truncating mutations in *TJP2* are shown to cause failure of protein localization, with disruption of tight junction structure [17, 37]. The resulting truncated proteins directly affect bile acid transport across the canalicular membrane, cause bile acid accumulation, hepatotoxicity and predispose to earlier progression towards cirrhosis, hepatocellular carcinoma and liver failure, more frequently compared to proteins with residual activity and resulting from missense mutations [46–49]. Thus, patients harboring severe genotypes are more likely to undergo LT [9,50,51].

Conversely, a dominant negative mechanism effect of pathogenic variants of *ATP8B1* rather than a loss-of- function mechanism is thought to determine an impairment in bile acid excretion. Similar native liver survival between patients harboring 2 protein-truncating mutations and patients harboring 1 or no protein-truncating mutations of *ATP8B1* was noted [8]. Here, we were unable to reliably investigate the PFIC1 subgroup effect due to the limited sample size.

It is important to emphasize that we only included observational studies that detailed the genotype and the LT outcome for each patient. For this reason, some works [5,7,40] that reported the LT outcome for the global sample of studied patients were not included. Another study limitation related to the small sample size is that we could not adopt the genotype categorization of the NAPPED consortium publications [32], which prevents us from assessing the effect on the LT outcome of different non-null genotype categories carrying only one or no predicted protein-truncating mutation. Moreover, we assumed that all alleles carrying missense variants are non-null. Indeed, the majority of the effects of the included missense variants are not studied by in vitro functional studies, the gold standard in predicting the effect on the protein. Some non-null genotypes like those carrying one p.Asp482Gly or p.Glu297Gly mutation and one protein-truncating mutation were found to be similar, regarding the severity of the disease course, to patients carrying two protein-truncating mutations [10]. This could introduce a bias of classification in which some patients with at least one missense mutation and classified as carrying non-null genotypes are actually harboring severe genotypes predisposing to liver dysfunction and indicating LT. In our study, sensitivity analysis by reclassifying *ABCB11* variants behaving as null variants did not reveal a difference in the overall effect size. However, the interpretation should be taken with caution because of the included studies’ small sample size.

Recently, new therapeutic drugs, like ileal bile acid transporter (IBAT) inhibitors, have revolutionized PFIC treatment by reducing toxic bile acids and delaying or preventing the need for LT [52]. These drugs act according to different protein dysfunction mechanisms, from transcription of the mutant PFIC gene to the processing and trafficking of the protein. To the best of our knowledge, except for gentamicin, which triggers translational read-through at premature stop codons and allows partial restoration of full-length protein synthesis, the effectiveness of the other therapeutic molecules likely requires some residual function of the protein [15,53]. Indeed, many patients, specifically those harboring truncating mutations, do not respond to novel drug therapies and still require surgical treatment, including LT before adulthood [10,52]. In this regard, studying the association between the PFIC genotype types and the need for LT helps find groups of patients harboring null genotypes, refractory to drug therapy, and candidates for earlier LT. Also, it helps find patients harboring non-null genotypes and candidates for early targeted drug therapy, before severe and irreversible liver damage.

### Strengths and limitations

The present study is characterized by several advantages. First, as far as we know, this is the first meta-analysis exploring the association between the genotype type and indication for LT.

Second, strict inclusion and exclusion criteria as well as evaluation by the NHLBI quality assessment tool were adopted in study selection to reduce artifacts of genotypes and LT outcomes misclassification. Third, we conducted different statistical tests to check for publication bias, as well as multiple sensitivity analyses to assess confounding and to improve the robustness of the results. Regarding statistical significance, this meta-analysis found an overall OR estimate of 2.79, indicating a positive association between the PFIC null genotype and the LT event. This association was consistent across the possible subgroup and sensitivity analyses. This result is useful to guide more personalized clinical decisions regarding patient selection for LT. However, our research had some limitations. First, all pooled studies were observational; thus, they were affected by biases of unmeasured confounding related to the modalities of the treatment before LT, comorbidities, and prognostic scores, as well as genetic and epigenetic-modifying factors [11] that could underestimate the total effect size. Second, very few studies, with small sample sizes and a lack of control groups, were available to be able to conduct PFIC4 subgroup analysis. Third, the validity of the sensitivity analyses of the studied confounders is uncertain due to missing data and the small number of pooled studies and patients in each subgroup.

Taking all the study’s limitations into account, we highlight the need for increasing sample sizes and integrating consortium and large datasets like NAPPED, which provide longitudinal data and clinical guidance [54], as well as the need for additional functional studies of PFIC pathogenic variants and controlled clinical trials including larger sample sizes across all PFIC subtypes. These would enable a better understanding of the impact of gene variants on the impairment of liver function and, robustly, comparing the frequencies of LT events between different genotype categories.

## Conclusions

This meta-analysis contributed to shedding more light on a potential link between types of genotypes in PFIC diseases and the indication of LT. Particularly, LT was found to be performed more frequently in patients harboring null genotypes. Furthermore, we underscore the importance of quantitatively synthesizing evidence related to types of genotypes and the intervention of LT for increasing the precision of the effect estimates and guiding decisions regarding personalized and early preventive LT before complications. This is particularly vital in developing countries with a high prevalence of PFIC diseases due to endogamy, in addition to the scarcity of donor livers and the need to prioritize the candidacy for LT.

## Supporting information

S1 Table PRISMA checklist. (DOCX)

S2 Table Quality assessment of the studies according to the NIH QAT. (DOCX)

S3 Table Patients’ genotypes and types. (DOCX)

S4 Table Extracted patient’s data. (XLSX)

S5 Fig Publication bias analysis using the leave-one-out methos. (TIF)

S6 Fig Sensitivity analysis stratifying articles according to age at last follow-up. (TIF)

S7 Fig Sensitivity analysis stratifying articles according to surgical intervention prior to LT. (TIF)

S8 Fig Sensitivity analysis stratifying articles according to study center/region. (TIF)

S9 Fig Sensitivity analysis following reclassification of borderline variants. (TIF)

## Abbreviations

ABCB11: ATP binding cassette subfamily B member 11
ATP8B1: ATPase Phospholipid Transporting 8B1
ABCB4: ATP Binding Cassette Subfamily B Member 4
BSEP: Bile salt export pump
C/D: cannot determine
CI: confidence interval
IBAT: ileal bile acid transporter
HGVS: Human Genome Variation Society
LT: liver transplantation
LT+: underwent liver transplantation
LT-: did not undergo liver transplantation
MDR3: Multidrug-resistance protein 3
MELD: Model for End-Stage Liver Disease
mo: Months
n: Sample size/number
N/A: Not applicable
NAPPED: Natural Course and Prognosis of PFIC and Effect of Biliary Diversion
NGS: Next-generation sequencing
NHLBI: National Heart, Lung, and Blood Institute
NR: not reported
PEBD: Partial external biliary diversion
PELD: Pediatric End-Stage Liver Disease
PFIC: Progressive familial intrahepatic cholestasis
PFIC1: Progressive familial intrahepatic cholestasis type 1
PFIC2: Progressive familial intrahepatic cholestasis type 2
PFIC3: Progressive familial intrahepatic cholestasis type 3
PFIC4: Progressive familial intrahepatic cholestasis type 4
RFLPA: Restriction Fragment Length Polymorphism Analysis
SSCPA: Single-Strand Conformation Polymorphism Analysis
TJP2: Tight Junction Protein 2
UDCA: Ursodeoxycholic acid
y: years
