# Supplementary material for: Types of genotypes in progressive familial intrahepatic cholestasis and liver transplantation: A meta-analysis of observational studies
Source: PLoS One. 2026 Jun 1;21(6):e0350508. doi: 10.1371/journal.pone.0350508 (PMC13225629; doi:10.1371/journal.pone.0350508)
Supplement: S2 Table — (DOCX) [file pone.0350508.s002.docx]

| **S2 Table. Quality assessment of the studies according to the NIH QAT.** | | | | | | | | | | | | | | | | |
| --- | --- | --- | --- | --- | --- | --- | --- | --- | --- | --- | --- | --- | --- | --- | --- | --- |
| **Cases series studies** | **Item** | |  |  |  |  |  |  |  |  |  |  |  |  |  |  |
| **Authors** | 1. Was the study question or objective clearly stated? | | 2. Was the study population clearly and fully described, including a case definition? | 3. Were the cases consecutive? | 4. Were the subjects comparable? | 5. Was the intervention clearly described? | 6. Were the outcome measures clearly defined, valid, reliable, and implemented consistently across all study participants? | 7. Was the length of follow-up adequate? | 8. Were the statistical methods well-described? | 9. Were the results well-described? | Quality Rating (Good, Fair, or Poor) |  |  |  |  |  |
| **Klomp et al.2000^[13]^** | Yes | | Yes | No | Yes | Yes | Yes | C/D | N/A | No | Fair |  |  |  |  |  |
| **Kang et al. 2019^[22]^** | Yes | | Yes | Yes | Yes | Yes | Yes | Yes | N/A | Yes | Good |  |  |  |  |  |
| **Zhang et al. 2020^[23]^** | Yes | | Yes | Yes | Yes | Yes | Yes | Yes | N/A | Yes | Good |  |  |  |  |  |
| **Lipi´nski et al.2021^[18]^** | Yes | | Yes | Yes | Yes | Yes | Yes | Yes | N/A | Yes | Good |  |  |  |  |  |
| **Chen et al. 2022^[24]^** | Yes | | Yes | Yes | Yes | Yes | Yes | Yes | Yes | Yes | Good |  |  |  |  |  |
| **Cohort studies** | | **Item** |  |  |  |  |  |  |  |  |  |  |  |  |  |  |
| **Authors** | | 1. Was the research question or objective in this paper clearly stated? | 2. Was the study populati-on clearly specified and defined? | 3. Was the participation rate of eligible persons at least 50%? | 4. Were all the subjects selected or recruited from the same or similar populations. Were inclusion and exclusion criteria for being in the study prespecifi-ed and applied uniformly to all participants? | 5. Was a sample size justificati-on, power descriptio-n, or variance and effect estimates provided? | 6. For the analyses in this paper, were the exposure (s) of interest measured prior to the outcome (s) being measured? | 7. Was the time-frame sufficient so that one could reasonably expect to see an association between exposure and outcome if it existed? | 8. For exposure-s that can vary in amount or level, did the study examine different levels of the exposure as related to the outcome? | 9. Were the exposure measures clearly defined, valid, reliable, and implement-ed consistentl-y across all study participants? | 10. Was the exposure (s) assessed more than once over time? | 11. Were the outcome measures clearly defined, valid, reliable, and implemented consistent-ly across all study participan-ts? | 12. Were the outcome assessors blinded to the exposure status of participan-ts? | 13. Was loss to follow-up after baselin-e 20% or less? | 14. Were key potential confoundi-ng variables measured and adjusted statisticall-y for their impact on the relationsh-ip between exposure (s) and outcome (s)? | Quality Rating (Good, Fair, or Poor) |
| **Jacquemin et al. 2001^[7]^** | | Yes | Yes | Yes | No | No | No | NR | No | No | No | No | No | Yes | No | Poor |
| **Chen et al. 2002^[21]^** | | Yes | Yes | Yes | Yes | No | No | Yes | Yes | Yes | No | Yes | Yes | Yes | No | Fair |
| **Giovannoni et al. 2015^[15]^** | | Yes | Yes | Yes | No | No | No | NR | Yes | Yes | No | No | Yes | Yes | No | Fair |
| **Jeyaraj et al.2021^[27]^** | | Yes | Yes | Yes | No | No | No | NR | Yes | Yes | No | Yes | Yes | NR | No | Fair |
| **Al-Hussaini et al. 2021^[28]^** | | Yes | Yes | Yes | Yes | No | No | Yes | Yes | Yes | Yes | Yes | Yes | Yes | No | Good |
| **Strautnieks et al. 2008^[30]^** | | Yes | Yes | Yes | Yes | No | No | Yes | Yes | Yes | Yes | Yes | Yes | Yes | No | Good |
| **Knisely et al. 2006^[29]^** | Yes | | No | Yes | NR | No | No | Yes | Yes | No | NR | Yes | Yes | Yes | No | Fair |
| **Davit-Spraul et al. 2010^[5]^** | Yes | | Yes | Yes | No | No | No | Yes | No | No | No | No | No | No | No | Poor |
| **Evason et al. 2011^[25]^** | | Yes | Yes | Yes | Yes | No | No | Yes | Yes | Yes | Yes | Yes | Yes | Yes | No | Good |
| **Colombo et al.2011^[14]^** | | Yes | Yes | Yes | Yes | No | No | Yes | Yes | Yes | Yes | Yes | Yes | Yes | No | Good |
| **Lipnski et al.2020^[17]^** | Yes | | Yes | Yes | Yes | No | No | Yes | Yes | Yes | No | Yes | Yes | Yes | No | Fair |
| **Shatz et al.2018^[16]^** | Yes | | Yes | Yes | No | No | NR | Yes | Yes | Yes | No | Yes | Yes | Yes | No | Fair |
| **Sambrotta et al. 2014^[26]^** | No | | Yes | Yes | No | No | No | Yes | Yes | Yes | Yes | Yes | Yes | No | No | Fair |
| **Hertel et al. 2021^[31]^** | Yes | | Yes | Yes | No | No | No | NR | No | No | NR | No | No | Yes | No | Poor |
| **Pfister et al.2022^[19]^** | Yes | | Yes | Yes | No | No | Yes | Yes | Yes | Yes | No | Yes | No | Yes | No | Fair |
| **Sahloul et al.2023^[20]^** | Yes | | Yes | Yes | No | No | Yes | Yes | Yes | Yes | Yes | Yes | No | No | No | Fair |
| C/D: cannot determine; N/A: not applicable; NR: not reported | | | | | | | | | | | | | | | | |
