# Supplementary material for: Types of genotypes in progressive familial intrahepatic cholestasis and liver transplantation: A meta-analysis of observational studies
Source: PLoS One. 2026 Jun 1;21(6):e0350508. doi: 10.1371/journal.pone.0350508 (PMC13225629; doi:10.1371/journal.pone.0350508)
Supplement: S3 Table — (DOCX) [file pone.0350508.s003.docx]

|  |  |  |  |  |  |
| --- | --- | --- | --- | --- | --- |
| **S3 Table. Patients genotypes and types.** | | | | | |
| **Type** | **Protein** | **Genotype** | **n cases** | **PFIC subtype/ gene** | **Study** |
| NN | p.Asp554Asn | Homozygous c.1660 G>A | 13 | PFIC1/ATP8B1 | **Klomp et al. 2000**^[13]^ |
| N | frameshift and protein truncation after codon 185 / [p.Gln1131*](https://www.ncbi.nlm.nih.gov/clinvar/variation/2736741/) | c.556-628del/ c.3391 C>T | 1 | PFIC1/ATP8B1 | **Chen et al. 2002**^[21]^ |
| NN | p.Arg296Cys | Homozygous c.886 C>T | 1 | PFIC1/ATP8B1 |  |
| NN | p.Ile694Asn | Homozygous c. 2081 T>A | 1 | PFIC1/ATP8B1 |  |
| NN | p.Val284Leu/ p.Ala382Alafs*16 | c.850G>C/ c.1145delC | 1 | PFIC2/ABCB11 |  |
| NN | p.Gly1004Asp | Homozygous 3137G>A | 1 | PFIC2/ABCB11 |  |
| NN | p.[Gly648[Valfs*6](https://mutalyzer.nl/normalizer/NM_003742.4(NP_003733.2):p.(Gly648Valfs*6))](https://www.ncbi.nlm.nih.gov/clinvar/variation/1453324/)/splicing | c.1941del/[c.2012-8T>G](https://www.ncbi.nlm.nih.gov/clinvar/variation/284637/) | 1 | PFIC2/ABCB11 | **Knisely et al. 2006**^[29]^ |
| N | Splicing/p.Ser25* | [c.2178+1G>A](https://www.ncbi.nlm.nih.gov/clinvar/variation/596620/)/c[.74C>A](https://www.ncbi.nlm.nih.gov/clinvar/variation/2734333/) | 1 | PFIC2/ABCB11 |  |
| NN | p.Asp482Gly/c.Arg1231Trp | c.1445A>G/c.3691C>T | 1 | PFIC2/ABCB11 |  |
| NN | p.Glu297Gly | Homozygous 890A>G | 1 | PFIC2/ABCB11 |  |
| NN | Splicing/p.Glu297Gly | [c.611+1G>A](https://www.ncbi.nlm.nih.gov/clinvar/variation/1526234/)/890A>G | 1 | PFIC2/ABCB11 |  |
| N | [p.Tyr472*](https://www.ncbi.nlm.nih.gov/clinvar/variation/950910/) | Homozygous c.1416T>A | 1 | PFIC2/ABCB11 |  |
| NN | p. Glu297Gly/splicing | c.890 A>G/[c.1435-13_1435-8del](https://www.ncbi.nlm.nih.gov/clinvar/variation/402331/) | 1 | PFIC2/ABCB11 |  |
| N | Splicing | Homozygous [c.2343+2T>C](https://www.ncbi.nlm.nih.gov/clinvar/variation/1458300/) | 1 | PFIC2/ABCB11 |  |
| N | [p.Tyr772*](https://www.ncbi.nlm.nih.gov/clinvar/variation/2679806/) | Homozygous c.2316T>G | 1 | PFIC2/ABCB11 |  |
| N | p.Thr127Hisfs*6 | Homozygous c.379delA | 3 | PFIC2/ABCB11 | **Strautnieks et al. 2008**^[30]^ |
| N | p.Tyr472* | Homozygous c.1416T>A | 1 | PFIC2/ABCB11 |  |
| N | p.Tyr772* | Homozygous c.2316T>G | 1 | PFIC2/ABCB11 |  |
| N | p.Arg1235* | Homozygous c.3703C>T | 1 | PFIC2/ABCB11 |  |
| N | splicing | Homozygous c.3213+1delG | 1 | PFIC2/ABCB11 |  |
| N | splicing | Homozygous c.390–1G>A | 2 | PFIC2/ABCB11 |  |
| N | splicing | Homozygous c.2343+2T>C | 1 | PFIC2/ABCB11 |  |
| N | splicing | Homozygous c.2611–2A>T | 1 | PFIC2/ABCB11 |  |
| N | p.0?/ splicing | Whole gene deletion/c.2179–2A>G | 1 | PFIC2/ABCB11 |  |
| N | p.0?/p.Arg575X | Whole gene deletion/c.1723C>T | 1 | PFIC2/ABCB11 |  |
| N | p.Leu380Trpfs*18/ p.Arg575* | c.1139delT/c.1723C>T | 1 | PFIC2/ABCB11 |  |
| N | p.Ala382_Ala388del/splicing | c.1145_1165del/c.2012–8T>G | 2 | PFIC2/ABCB11 |  |
| N | p.Gly648Valfs*6/splicing | c.1941delA/c.2012–8T>G | 1 | PFIC2/ABCB11 |  |
| N | p.Arg575*/p.Arg1090* | c.1723C>T/c.3268C>T | 1 | PFIC2/ABCB11 |  |
| N | p.Ser25*/splicing | c.74C>A/c.2178-1G>A | 1 | PFIC2/ABCB11 |  |
| N | p.Arg575*/splicing | c.1723C>T/c.2178+1G>T | 1 | PFIC2/ABCB11 |  |
| N | splice defect/p.Glu1302* | c.2178+1G>A/c.3904G>T | 1 | PFIC2/ABCB11 |  |
| N | splicing/p.Glu1302* | c.611+1G>A/c.3904G>T | 1 | PFIC2/ABCB11 |  |
| NN | p.Arg303Lys | Homozygous c.908G>A | 1 | PFIC2/ABCB11 |  |
| NN | p.Arg470Gln | Homozygous c.1409G>A | 1 | PFIC2/ABCB11 |  |
| NN | p.Arg487Pro | Homozygous c.1460G>C | 1 | PFIC2/ABCB11 |  |
| NN | p.Ile541Leu | Homozygous c.1621A>C | 2 | PFIC2/ABCB11 |  |
| NN | p.Ala570Thr | Homozygous c.1708G>A | 1 | PFIC2/ABCB11 |  |
| NN | p.Gly758Arg | Homozygous c.2272G>C | 1 | PFIC2/ABCB11 |  |
| NN | p.Gly766Arg | Homozygous c.2296G>A | 1 | PFIC2/ABCB11 |  |
| NN | p.Arg832Cys | Homozygous c.2494C>T | 1 | PFIC2/ABCB11 |  |
| NN | p.Asn979Asp | Homozygous c.2935A>G | 1 | PFIC2/ABCB11 |  |
| NN | p.Gly982Arg | Homozygous c.2944G>A | 1 | PFIC2/ABCB11 |  |
| NN | p.Arg1128Cys | Homozygous c.3382C>T | 3 | PFIC2/ABCB11 |  |
| NN | p.Arg1153His | Homozygous c.3458G>A | 2 | PFIC2/ABCB11 |  |
| NN | p.Thr1210Pro | Homozygous c.3628A>C | 1 | PFIC2/ABCB11 |  |
| NN | p.Lys930Glufs*79/p.Val481Glu | c.2787_2788insGAGAT/c.1442T>A | 2 | PFIC2/ABCB11 |  |
| NN | p.Arg575*/p.Gly982Arg | c.1723C>T/c.2944G>A | 1 | PFIC2/ABCB11 |  |
| NN | p.Thr463Ile/p.Gln1215* | c.3643C>T/c.1388C>T | 1 | PFIC2/ABCB11 |  |
| NN | Splicing/p.Ile541Leu | c.908+1delG/c.1621A>C | 1 | PFIC2/ABCB11 |  |
| NN | Splicing/p.Arg1153His | c.2012-8T>G/c.3458G>A | 1 | PFIC2/ABCB11 |  |
| NN | p. Thr242Ile/Splicing | c.725C>T/c.2178+1G>A | 1 | PFIC2/ABCB11 |  |
| NN | splicing/p.Arg948Cys | c.2178+1G>A/c.2842C>T | 1 | PFIC2/ABCB11 |  |
| NN | splicing/p.Thr859Arg | c.2178+1G>A/c.2576C>G | 1 | PFIC2/ABCB11 |  |
| NN | splicing /p.Ile541Thr | c.2343+1G>T/c.1622T>C | 1 | PFIC2/ABCB11 |  |
| NN | p.Leu1242Ile/p.Leu413Trp | c.1238T>G/c.3724C>A | 2 | PFIC2/ABCB11 |  |
| NN | p.Tyr472Cys/p.Ile512Thr | c.1415A>G/c.1535T>C | 2 | PFIC2/ABCB11 |  |
| NN | p.Ala1110Glu/p.Leu50Ser | c.149T>C/c.3329C>A | 2 | PFIC2/ABCB11 |  |
| NN | p.Arg1153His/p.Arg1231Gln | c.3458G>A/c.3692G>A | 1 | PFIC2/ABCB11 |  |
| NN | p.Tyr157Cys /p.Gly1298Arg | c.470A>G /c.3892G>A | 1 | PFIC2/ABCB11 |  |
| NN | p.Gly327Glu/ p.Ser1154Pro | c.980G>A/c.3460T>C | 1 | PFIC2/ABCB11 |  |
| NN | p.Glu297Gly/p.0? | c.890A>G/Total gene deletion | 1 | PFIC2/ABCB11 |  |
| NN | p.Glu297Gly/ p.Val368Argfs*27 | c.890A>G/c.1101_1102delAG | 1 | PFIC2/ABCB11 |  |
| NN | p.Glu297Gly/ p.Ile528Serfs*21 | c.890A>G/c.1583_1584delTA | 3 | PFIC2/ABCB11 |  |
| NN | p.Glu297Gly/ p.Gly648Valfs*6 | c.890A>G/c.1941delA | 1 | PFIC2/ABCB11 |  |
| NN | p.Glu297Gly/ p.Lys969_Lys972del | c.890A>G/c.2906_2917del | 2 | PFIC2/ABCB11 |  |
| NN | p.Glu297Gly/ p.Val1147* | c.890A>G/c.3438delA | 1 | PFIC2/ABCB11 |  |
| NN | p.Glu297Gly/ p.Val1164Glyfs*7 | c.890A>G/c.3491delT | 1 | PFIC2/ABCB11 |  |
| NN | p.Glu297Gly/ p.Arg520X* | c.890A>G/c.1558A>T | 1 | PFIC2/ABCB11 |  |
| NN | p.Glu297Gly/ p.Arg1090* | c.890A>G/c.3268C> T | 1 | PFIC2/ABCB11 |  |
| NN | p.Glu297Gly/ p.Glu1302* | c.890A>G/c.3904G>T | 1 | PFIC2/ABCB11 |  |
| NN | p.Glu297Gly/ splicing | c.890A>G/c.611+1G>A | 2 | PFIC2/ABCB11 |  |
| NN | p.Glu297Gly/ splicing | c.890A>G/c.1435-13_1435-8del | 1 | PFIC2/ABCB11 |  |
| NN | p.Glu297Gly/ splicing | c.890A>G/c.2012-8T>G | 2 | PFIC2/ABCB11 |  |
| NN | p.Glu297Gly/ splicing | c.890A>G/ c.2343+1G>T | 3 | PFIC2/ABCB11 |  |
| NN | p.Glu297Gly/p.Ala390Pro | c.890A>G/ c.1168G>C | 1 | PFIC2/ABCB11 |  |
| NN | p.Glu297Gly/p.Phe548Tyr | c.890A>G/ c.1643T>A | 2 | PFIC2/ABCB11 |  |
| NN | p.Glu297Gly/ p.Ala588Val | c.890A>G/ c.1763C>T | 1 | PFIC2/ABCB11 |  |
| NN | p.Glu297Gly/ p.Arg948Cys | c.890A>G/ c.2842C>T | 1 | PFIC2/ABCB11 |  |
| NN | p.Glu297Gly/ p.Ala1110Glu | c.890A>G/ c.3329C>A | 1 | PFIC2/ABCB11 |  |
| NN | p.Glu297Gly/ p.Arg1153Cys | c.890A>G/ c.3457C>T | 1 | PFIC2/ABCB11 |  |
| NN | p.Glu297Gly | Homozygous c.890A>G | 8 | PFIC2/ABCB11 |  |
| NN | p.Glu297Gly/ p.Asp482Gly | c.890A>G/c.1445A>G | 2 | PFIC2/ABCB11 |  |
| NN | p.Asp482Gly | Homozygous c.1445A>G | 9 | PFIC2/ABCB11 |  |
| NN | p.Asp482Gly/ p.Arg520* | c.1445A>G/c.1558A>T | 1 | PFIC2/ABCB11 |  |
| NN | p.Asp482Gly/ splicing | c.1445A>G/c.908+1G>A | 1 | PFIC2/ABCB11 |  |
| NN | p.Asp482Gly/ p.Arg313Ser | c.1445A>G/c.937C>A | 1 | PFIC2/ABCB11 |  |
| NN | p.Asp482Gly/ p.Gly410Asp | c.1445A>G/c.1229G>A | 1 | PFIC2/ABCB11 |  |
| NN | p.Asp482Gly/ p.Arg470Gln | c.1445A>G/c.1409G>A | 1 | PFIC2/ABCB11 |  |
| NN | p.Asp482Gly/ p.Asn515Thr | c.1445A>G/ c.1544A>C | 4 | PFIC2/ABCB11 |  |
| NN | p.Asp482Gly/ p.Arg517His | c.1445A>G/c.1550G>A | 1 | PFIC2/ABCB11 |  |
| NN | p.Asp482Gly/ p.Gly562Asp | c.1445A>G/c.1685G>A | 1 | PFIC2/ABCB11 |  |
| NN | p.Asp482Gly/ p.Ala588Val | c.1445A>G/c.1763C>T | 1 | PFIC2/ABCB11 |  |
| NN | p.Asp482Gly/ p.Arg832Cys | c.1445A>G/c.2494C>T | 1 | PFIC2/ABCB11 |  |
| NN | p.Asp482Gly/ p.Thr1029Lys | c.1445A>G/c.3086C>A | 1 | PFIC2/ABCB11 |  |
| NN | p.Asp482Gly/ p.Arg1231Trp | c.1445A>G/c.3691C>T | 1 | PFIC2/ABCB11 |  |
| NN | p.Glu297Gly | Homozygous c.890A>G | 1 | PFIC2/ABCB11 | **Evason et al. 2011**^[25]^ |
| N | p.Arg575*/splicing | c.1723C>T/c.2178+1G>T | 1 | PFIC2/ABCB11 |  |
| NN | p.Ala570T/p.Val1212Phe | c.1708G>A/ c.3634G>T | 1 | PFIC2/ABCB11 |  |
| NN | p.Leu1055Pro | Homozygous c.3164T>C | 1 | PFIC2/ABCB11 |  |
| NN | p.Arg1231Gln/ p.Gly766Arg | c.3692G>A/ c.2296G>A | 1 | PFIC2/ABCB11 |  |
| N | p.Arg928*/ p.Arg1090* | c.2782C>T /c.3268C>T | 1 | PFIC2/ABCB11 |  |
| NN | p.Gly1116Glu/ splicing | c.3347G>A /IVS 23-8 G>A | 1 | PFIC2/ABCB11 |  |
| NN | p.Gly982Arg/ p.Gly766Arg | c.2944G>A /c.2296G>A | 2 | PFIC2/ABCB11 |  |
| NN | p.Cys107Arg/ splicing | c.319T>C /c.611+4A>G | 1 | PFIC2/ABCB11 |  |
| N | p.Arg575*/ splicing | c.1723C>T/c.2178+1G>T | 1 | PFIC2/ABCB11 |  |
| NN | p.Thr175Ala /p.Phe35Leu+ p.Thr775Met | c.523A>G/c.1069T>C +c.2324C>T | 1 | PFIC3/ABCB4 | **Colombo et al. 2011**^[14]^ |
| NN | p.Ser379Lysfs*413/ p.Leu701Pro | c.1135_1136insAA/c.2102T>C | 1 | PFIC3/ABCB4 |  |
| NN | p.Glu888*/p.Ala250Pro + p.Met630Val | c.2662G>T/c.748G>C + c.1888A>G | 1 | PFIC3/ABCB4 |  |
| NN | p.Ala286Val/ p.Ser320Phe | c.857C>T /c.959C>T | 1 | PFIC3/ABCB4 |  |
| NN | p.Gly126Glu/p.Ala511Thr | c.377G>A/c.1531G>A | 2 | PFIC3/ABCB4 |  |
| NN | p.Pro726Thr/p.Arg590Gln + p.Gly762* | c.2176C>A/c.1769G>A + c.2284G>T | 1 | PFIC3/ABCB4 |  |
| NN | p.Ala364Val /p.Ala737Val | c.1091C>T/c.2210C>T | 1 | PFIC3/ABCB4 |  |
| NN | p.Val475Ala/p.Ala840Asp | c.1424T>C/c.2519C>A | 1 | PFIC3/ABCB4 |  |
| NN | p.Glu558Lys/p.Gly723Glu+ p.Ala1193Thr | c.1672G>A/c.2168G>A + c.3577G>A | 2 | PFIC3/ABCB4 |  |
| NN | p.Gly954Ser | Homozygous c.2860G>A | 1 | PFIC3/ABCB4 |  |
| NN | p.Tyr279*/p.Ser320Phe | c.959C>T/c.837T>A | 1 | PFIC3/ABCB4 |  |
| N | p.Leu724Alafs*744 | Homozygous c.2169_2170insG | 2 | PFIC3/ABCB4 |  |
| NN | p.Tyr403His | Homozygous c.1207T>C | 1 | PFIC3/ABCB4 |  |
| NN | p.Gly70Arg + p.Arg590Gln/p.Ser320Phe | c.208G>C + c.1769G>A/ c.959C>T | 3 | PFIC3/ABCB4 |  |
| NN | p.Ser320Phe | Homozygous c.959C>T | 1 | PFIC3/ABCB4 |  |
| N | p.Ala256Thrfs*54 | Homozygous c.766_769delGCCT | 1 | PFIC4/TJP2 | **Sambrotta et al.2014**^[26]^ |
| N | p.Ser296Alaf*15 | Homozygous c.885delC | 2 | PFIC4/TJP2 |  |
| N | p.Tyr261Serfs*50 | Homozygous c.782delA | 1 | PFIC4/TJP2 |  |
| N | p.Ala454Glyfs*60 | Homozygous c.1361delC | 2 | PFIC4/TJP2 |  |
| N | p.Arg664Serfs*2 | Homozygous c.1992-2A>G | 2 | PFIC4/TJP2 |  |
| N | p.Glu318Glyfs*2 | Homozygous c.953-735_2356-249del | 2 | PFIC4/TJP2 |  |
| N | p.Ser1136Argfs*2 | Homozygous c.3408-?_3573+?del | 1 | PFIC4/TJP2 |  |
| N | p.Arg632* | Homozygous c.1894C>T | 1 | PFIC4/TJP2 |  |
| NN | p.Gly446Arg | Homozygous c.1336G>A | 1 | PFIC1/ATP8B1 | **Giovannoni et al. 2015**^[15]^ |
| N | p.Arg930* | Homozygous c.2788C>T | 1 | PFIC1/ATP8B1 |  |
| N | splicing/p.Arg1014* | c.2097+2T>C/c.3040C>T | 1 | PFIC1/ATP8B1 |  |
| N | p.Trp1095* | Homozygous c.3284G>A | 1 | PFIC1/ATP8B1 |  |
| N | p.Met320Valfs*13/- | c.del958_967fs*14/ del5'UTR-ex18 | 1 | PFIC1/ATP8B1 |  |
| N | p.His609Hisfs*46 | Homozygous c.1822_1823insCA | 1 | PFIC2/ABCB11 |  |
| N | p.Tyr304* | Homozygous c.912T>G | 1 | PFIC2/ABCB11 |  |
| N | pGlu135Lys*/ p.L1099LfsX38 | c.403G>A*/ c.3297delC | 1 | PFIC2/ABCB11 |  |
| NN | p.Arg1153His/ p.Arg1050Cys | c.3458G>A/c.3148C>T | 1 | PFIC2/ABCB11 |  |
| NN | p.Arg832Cys | Homozygous c.2494C>T | 1 | PFIC2/ABCB11 |  |
| NN | p.Ile669Val/ p.Ala570Thr | c.2005A>G/c.1708G>A | 1 | PFIC2/ABCB11 |  |
| NN | p.Ile541Leu | Homozygous c.1621A>C | 2 | PFIC2/ABCB11 |  |
| NN | p.Thr625Asnfs*5/p.Ala570Thr | c.1873insA/c.1708G>A | 1 | PFIC2/ABCB11 |  |
| NN | p.Ala570Val | Homozygous c.1709C>T | 1 | PFIC2/ABCB11 |  |
| NN | p.Arg52Trp/p.His615Arg | c.154C>T/c.1844A>G | 1 | PFIC2/ABCB11 |  |
| NN | p.Lys93Glufs*49/p.Arg1153Cys | c.2787_2788insGAGAT/c.3457C>T | 1 | PFIC2/ABCB11 |  |
| NN | p.Arg948Cys/ p.Ser1027Arg | c.2842C>T /c.3081T>A | 2 | PFIC2/ABCB11 |  |
| NN | p.Asp482G | Homozygous c.1445A>G | 1 | PFIC2/ABCB11 |  |
| NN | p.Arg470Gln | Homozygous c.1409G>A | 2 | PFIC2/ABCB11 |  |
| N | p.Arg595* | Homozygous c.1783C>T | 1 | PFIC3/ABCB4 |  |
| N | p.Arg595*/ p.Ala313Metfs*72 | c.1783C>T/ c.937_992ins/del6 | 1 | PFIC3/ABCB4 |  |
| NN | p.Leu481Arg/ p.Arg652Gly | c.1442T>G/ c.1954A>G | 1 | PFIC3/ABCB4 |  |
| NN | p.Pro726Leu | Homozygous c.2177C>T | 6 | PFIC3/ABCB4 | **Shatz et al.2018**^[16]^ |
| NN | p.Gly722Ala/ splicing | c.2165G>C/ c.348615G>A | 1 | PFIC3/ABCB4 |  |
| NN | p.Arg590Gln | Homozygous c.1769G>A | 1 | PFIC3/ABCB4 |  |
| NN | p.Glu528Asp/ p.Arg652Gly | c.1584G>C/ c.1954A>G | 1 | PFIC3/ABCB4 |  |
| NN | p.Thr175Ala/ p.Arg652Gly | c.523A>G/ c.1954A>G | 1 | PFIC3/ABCB4 |  |
| NN | p.Leu23Hisfs*16/ p.Ser27Gly | c.67-68insAC/c.79A>G | 1 | PFIC3/ABCB4 |  |
| N | splicing | Homozygous c.3633+1G>T | 2 | PFIC3/ABCB4 |  |
| NN | p.Ala953Asp | Homozygous c.2858C>A | 2 | PFIC3/ABCB4 |  |
| NN | splicing/ p.Gln1181Glu | c.286+1G>A/ c.3541C>G | 1 | PFIC3/ABCB4 |  |
| NN | p.Ala953Asp/ p.Asn168Asn | c.2858C>A/c.504C>T | 1 | PFIC3/ABCB4 |  |
| NN | p.Ala794Pro | Homozygous c.2380G>C | 1 | PFIC3/ABCB4 |  |
| NN | p.Asn168Asn/p.Thr175Ala | c.504C>T/c.523A>G | 1 | PFIC3/ABCB4 |  |
| N | splicing | Homozygous c.2783+1G>C | 1 | PFIC3/ABCB4 |  |
| NN | p.Tyr1086Ser | Homozygous c.3257A>C | 1 | PFIC3/ABCB4 |  |
| NN | p.Pro95Ser | Homozygous c.283C>T | 2 | PFIC3/ABCB4 |  |
| NN | p.Ser27Gly/ p.Ser320Phe | c.79A>G/c.959C>T | 2 | PFIC3/ABCB4 |  |
| NN | p.Glu636Gly/ p.Ala865Val | c.1907A > G / c.2594C>T | 1 | PFIC2/ABCB11 | **Kang et al. 2019**^[22]^ |
| N | p.Tyr472* | Homozygous c.1416T>A | 1 | PFIC2/ABCB11 |  |
| NN | p.Glu585*/p.Leu749Pro | c.1753G > T/ c.2246T>C | 1 | PFIC1/ATP8B1 |  |
| NN | p.Gly446R/p.Phe529del | c.1336 G>A/ c.1587_1589delCTT | 1 | PFIC1/ATP8B1 | **Zhang et al 2020**^[23]^ |
| N | p.Gln794X/p.Arg1057* | c.2380C>T/c.3170C>T | 1 | PFIC2/ABCB11 |  |
| NN | p.Arg470Hisfs*8/p.Gly1004Asp | c.1407delG/c.3011G>A | 1 | PFIC2/ABCB11 |  |
| NN | p.Ala588Val | Homozygous c.1763C>T | 1 | PFIC2/ABCB11 |  |
| NN | p.Ile1170Thr | Homozygous c.3509T>C | 1 | PFIC2/ABCB11 |  |
| N | p.Thr830Asnfs*11/p.Ala1047Profs*8 | c.2489insA/c.3139_3141delGCAins CC | 1 | PFIC3/ABCB4 |  |
| NN | p.Gly319Arg/p.Gly1074Arg | c.955G>C/c.3220G>A | 1 | PFIC3/ABCB4 |  |
| NN | p.Pro479Lys | Homozygous c.1436C>T | 1 | PFIC3/ABCB4 |  |
| NN | p.Asn1048Thrfs*8/p.Ala1047Pro | c.3143delA/c.3139G>C | 1 | PFIC3/ABCB4 |  |
| NN | p.Asp496Val/p.Ile541Leu | c.1487A>T/c.1621A>C | 1 | PFIC2/ABCB11 | **Lipi’nski et al. 2020**^[17]^ |
| NN | p.Asp482Gly/p.Arg520* | c.1445A>G/c.1558A>T | 1 | PFIC2/ABCB11 |  |
| NN | p. Leu308Phe/p.Asp482Gly/p.Met1202Thr | c.922C>T/c.1445A>G/c.3605T>C | 1 | PFIC2/ABCB11 |  |
| N | splicing/splicing | Homozygous c.150+3A>G | 1 | PFIC2/ABCB11 |  |
| NN | p.Pro474Leu | Homozygous c.1421C>T | 1 | PFIC2/ABCB11 |  |
| NN | p.Asp482Gly | Homozygous c.1445A>G | 1 | PFIC2/ABCB11 |  |
| NN | p.Met62Lys/p.Asn515Thr | c.185T>A/c.1544A>C | 1 | PFIC2/ABCB11 |  |
| NN | p.Gln361*/p.Asp482Gly | c.1081C>T/c.1445A>G | 1 | PFIC2/ABCB11 |  |
| NN | p.Ser320Phe/splicing | c.959C>T/c.1119+1G>A | 1 | PFIC3/ABCB4 |  |
| NN | p.Arg582Gln/p.Cys717Ser | c.1745G>A /c.2149T>A | 1 | PFIC3/ABCB4 |  |
| NN | p.Val69Met/p.Arg367* | c.205G>A/c.1099C>T | 1 | PFIC4/TJP2 |  |
| N | [p.Arg680Glufs*4](https://mutalyzer.nl/normalizer/NM_003742.4(NP_003733.2):p.(Arg680Glufs*4)) | Homozygous c.2037dup | 1 | PFIC4/TJP2 |  |
| NN | p.Gln415Arg | Homozygous c.1244A>G | 1 | PFIC1/ATP8B1 | **Jeyaraj et al. 2021**^[27]^ |
| NN | p.Thr456Met/p.Glu695Lys | c.1367C>T/c.2083G>A | 1 | PFIC1/ATP8B1 |  |
| N | p.Arg930* | Homozygous c.2788C>T | 1 | PFIC1/ATP8B1 |  |
| NN | p.Ile245Thrfs*26/p.Gly260Asp | c.731_732insA/c.779G>A | 1 | PFIC2/ABCB11 |  |
| NN | p.Gln361*/p.Asp482Gly | c.1081C>T/ c.1445A>G | 1 | PFIC2/ABCB11 |  |
| NN | p.Arg470Gln | Homozygous c.1409G>A | 2 | PFIC2/ABCB11 |  |
| N | p.Tyr472* | Homozygous c.1416T>A | 1 | PFIC2/ABCB11 |  |
| NN | p.Asp482Gly | Homozygous c.1445A>G | 1 | PFIC2/ABCB11 |  |
| NN | p.Tyr1311*/p.Met559Thr | c.1676T>C/c.3933C>G | 1 | PFIC2/ABCB11 |  |
| NN | p.Val903Gly | Homozygous c.2708T>G | 1 | PFIC2/ABCB11 |  |
| NN | p.Asn1173Asp/p.Thr1210Phe | c.3517A>G/c.3628A>C | 1 | PFIC2/ABCB11 |  |
| N | p.Glu1302* | Homozygous c.3904G>T | 1 | PFIC2/ABCB11 |  |
| N | splicing | Homozygous c.1230+1G>T | 1 | PFIC3/ABCB4 |  |
| NN | p.Ala542Pro | Homozygous c.1624G>C | 1 | PFIC3/ABCB4 |  |
| NN | p.Pro551Leu | Homozygous c.1652C>T | 1 | PFIC3/ABCB4 |  |
| NN | p.Lys620del | Homozygous c.1858_1860delAAG | 1 | PFIC3/ABCB4 |  |
| N | splicing | Homozygous c.698+1 G>T | 1 | PFIC1/ATP8B1 | **Al Hussaini et al.2021**^[28]^ |
| NN | p.Arg412Cys | Homozygous c.1234C>T | 1 | PFIC1/ATP8B1 |  |
| N | p.lle129Thrfs*38 | Homozygous c.386_390delTCTTA | 1 | PFIC1/ATP8B1 |  |
| NN | p.Arg832Cys | Homozygous c.2494C>T | 4 | PFIC2/ABCB11 |  |
| N | p.Thr127Hisfs*6 | Homozygous c.379delA | 14 | PFIC2/ABCB11 |  |
| NN | p.Arg1153Cys | Homozygous c.3457C>T | 6 | PFIC2/ABCB11 |  |
| NN | p.Arg1153Cys + p.Val444AIa | Homozygous c.3457C>T + c.1331T>C | 1 | PFIC2/ABCB11 |  |
| NN | p.Arg1128Cys + p.Gln312His | Homozygous c.3382C>T + c.936 G>T | 2 | PFIC2/ABCB11 |  |
| NN | p.Gly1004Asp | Homozygous c.3011G>A | 1 | PFIC2/ABCB11 |  |
| NN | p.Glu636Gly | Homozygous c.1907A>G | 1 | PFIC2/ABCB11 |  |
| N | p.Tyr1208* | Homozygous c.3624T>A | 1 | PFIC2/ABCB11 |  |
| N | splicing | Homozygous c.611+5G>A | 1 | PFIC2/ABCB11 |  |
| NN | p.Glu898Lys | Homozygous c.2692G>A | 8 | PFIC3/ABCB4 |  |
| NN | p.Met74Arg | Homozygous c.221T>G | 1 | PFIC3/ABCB4 |  |
| NN | p.Arg969His | Homozygous c.2906G>A | 3 | PFIC3/ABCB4 |  |
| NN | splicing/p.Glu528Asp | c.456G>A/c.1584G>C | 1 | PFIC3/ABCB4 |  |
| NN | p.Phe970Leu | Homozygous c.2908T>C | 1 | PFIC3/ABCB4 |  |
| NN | p.Arg176Trp | Homozygous c.526C>T | 4 | PFIC3/ABCB4 |  |
| NN | p.Ser99Phe | Homozygous c.296C>T | 2 | PFIC3/ABCB4 |  |
| NN | p.Ile460Phe | Homozygous c.1378A>T | 1 | PFIC3/ABCB4 |  |
| NN | splicing (r.spl?) | Homozygous c.2064+G>C | 1 | PFIC3/ABCB4 |  |
| N | p.Phe210Serfs*5 | Homozygous c.628_643del | 3 | PFIC3/ABCB4 |  |
| NN | p.Cys717Ser/ p.Arg582Gln | c.2149T>A/c.1745G>A | 1 | PFIC3/ABCB4 | **Lipi’nski et al. 2021**^[18]^ |
| NN | p.Leu1182His | Homozygous c.3524T>A | 1 | PFIC3/ABCB4 |  |
| NN | p.Ser320Phe/splicing | c.959C>T/c.1119+1G>A | 1 | PFIC3/ABCB4 |  |
| NN | p.Met301Lys/splicing | c.902T>A /c.3279+1G>A | 1 | PFIC3/ABCB4 |  |
| NN | p.Arg928Gly/splicing | c.2782A>G/c.136-2A>G | 1 | PFIC3/ABCB4 | **Chen et al. 2022**^[24]^ |
| NN | p.Ala601Thr/p.Arg469Lys | c.1801G>A/c.1406G>A | 1 | PFIC3/ABCB4 |  |
| N | splicing/p.Ile1034Asnfs*4 | c.80+1G>C/c.3100_3101insA | 1 | PFIC3/ABCB4 |  |
| NN | p.Asp972Asn/p.Leu322Pro | c.2914G>A/c.965T>C | 1 | PFIC3/ABCB4 |  |
| N | p.Trp708*/p.Gly1264Alafs*38 | c.2123G>A/c.3789delA | 1 | PFIC3/ABCB4 |  |
| N | p.Arg1057*/splicing | c.3169C>T/ c.2178+1G>A | 1 | PFIC2/ABCB11 | **Pfister et al. 2023**^[19]^ |
| N | p.Arg8* | Homozygous c.22C> T | 1 | PFIC2/ABCB11 |  |
| NN | p.Asp590Glu | Homozygous c.1770C>A | 1 | PFIC2/ABCB11 |  |
| NN | p.Glu297Gly | Homozygous c.890A>G | 4 | PFIC2/ABCB11 |  |
| NN | p.Glu297Gly/p.Asp482Gly | c.890A>G/c.1445A>G | 1 | PFIC2/ABCB11 |  |
| N | p.Arg415* | Homozygous c.1243C>T | 1 | PFIC2/ABCB11 |  |
| NN | p.Gly260Asp/p.Gly877Arg | c.779G>A/c.2629G>A | 1 | PFIC2/ABCB11 |  |
| NN | p.Arg8*/p.Ala570Val/p.Ile1669Val | c.22C>T/c.1709C>T/c.2005A>G | 1 | PFIC2/ABCB11 |  |
| NN | p.Asp482Gly | Homozygous c.1445A>G | 3 | PFIC2/ABCB11 |  |
| N | p.Ser1154Glnfs*17/p.Val1164Glyfs*7 | c.3459_3460delCTinsG/c.3491delT | 1 | PFIC2/ABCB11 |  |
| NN | p.Gly873Ser | Homozygous c.2617G> A | 1 | PFIC2/ABCB11 |  |
| NN | p.Ser462Arg/p.Ile879Arg | c.1384A>C/c.2636T>G | 1 | PFIC2/ABCB11 |  |
| N | p.Arg1235* | Homozygous c.3703C>T | 1 | PFIC2/ABCB11 |  |
| NN | p.Tyr721Cys/p.Ala1028Ala | c.2162A>G/c.3084A>G | 1 | PFIC2/ABCB11 |  |
| NN | p.Asn1173Asp | Homozygous c.3517A> G | 1 | PFIC2/ABCB11 |  |
| NN | p.Arg387His/p.Ser901Arg | c.1160G>A/c.2703C>G | 1 | PFIC2/ABCB11 |  |
| NN | p.Glu297Gly/p.Asp482Gly | c.890A>G/c.1445A>G | 3 | PFIC2/ABCB11 |  |
| NN | p.Ala384Pro | Homozygous c.1150G > C | 1 | PFIC2/ABCB11 |  |
| NN | splicing/p.Met677Val | c.2178+1G>A/c.2029A>G | 1 | PFIC2/ABCB11 |  |
| NN | p.Leu656Alafs*9/p.Glu297Gly | c.890A>G/c.1966_1967delTT | 1 | PFIC2/ABCB11 |  |
| NN | p.Ser462Arg/p.Ile879Arg | c.1384A>C/c.2636T>G | 1 | PFIC2/ABCB11 |  |
| NN | p.Arg1128Cys/p.Leu1165del | c.3382C>T/c.3495_3497del | 1 | PFIC2/ABCB11 |  |
| NN | splicing/p.Glu297Gly | c.99-1G>T/c.890A>G | 1 | PFIC2/ABCB11 |  |
| NN | p.Gly1118Ser/p.Thr1121Ile | c.3352G>A/c.3362C>T | 1 | PFIC2/ABCB11 |  |
| NN | p.Arg571Thr/p.Arg928* | c.1712G>C/c.2782C> T | 1 | PFIC2/ABCB11 |  |
| NN | p.Leu1165del | Homozygous c.3495_3497del | 1 | PFIC2/ABCB11 |  |
| N | p.Ile610Glnfs*45 | Homozygous c.1827_1828insCA | 1 | PFIC2/ABCB11 |  |
| NN | p.Arg571Thr/p.Arg928* | c.1712G>C/c.2782C> T | 1 | PFIC2/ABCB11 |  |
| NN | p.Arg1235*/p.Arg378His | c.3703C>T/c.1160G>A | 1 | PFIC2/ABCB11 |  |
| NN | p.Gly255Glu/p.Arg487Pro | c.1460G>C/c.764G> A | 1 | PFIC2/ABCB11 |  |
| NN | p.?/p.Arg378His | c.-27-789_99-48del/ c.1160G>A | 1 | PFIC2/ABCB11 |  |
| NN | p.Ala384Pro | Homozygous c.1150G>C | 1 | PFIC2/ABCB11 |  |
| NN | p.Leu1165del | Homozygous c.3495_3497del | 1 | PFIC2/ABCB11 |  |
| NN | p.Glu297Gly | Homozygous c.890A>G | 2 | PFIC2/ABCB11 |  |
| NN | p.Asp482Gly/p.Ile420Asn | c.1445A>G/c.1259T>A | 1 | PFIC2/ABCB11 |  |
| NN | p.Ile516Met/p.Asn1173Asp | c.1548T>G/c.3517A>G | 1 | PFIC2/ABCB11 |  |
| NN | p.Arg487His/p.Arg487Pro | c.1460G>A/c.1460G>C | 1 | PFIC2/ABCB11 |  |
| NN | p.Tyr93*/p.Ala1028Glu | c.279C>A/c.3083_3084delinsAG | 1 | PFIC2/ABCB11 |  |
| NN | p.Lys455Asn | Homozygous c.1365G>T | 1 | PFIC1/ATP8B1 | **Sahloul et al. 2023**^[20]^ |
| N | splicing | Homozygous c.2097 + 2T > C | 2 | PFIC1/ATP8B1 |  |
| NN | p.Gly260Asp/p.Glu297Gly | [c.779G>A](https://mutalyzer.nl/normalizer/NM_003742.2:c.(779G%3EA))/c.890A>G | 1 | PFIC2/ABCB11 |  |
| NN | p.Asn1173Asp/p.Thr1210Pro | c.3517A>G/c.3628A>C | 1 | PFIC2/ABCB11 |  |
| NN | p.Asp496Val/ splicing | [c.1487A>T](https://mutalyzer.nl/normalizer/NM_003742.2:c.(1487A%3ET))/ c.2178 + 1G>A | 1 | PFIC2/ABCB11 |  |
| NN | p.Ile213Thr/p.Asp482Gly | c.638T>C/c.1445A>G | 1 | PFIC2/ABCB11 |  |
| NN | p.Ser462Arg/p.Ile879Arg | -/ c.2636T>G | 2 | PFIC2/ABCB11 |  |
| NN | p.Ile333Phe/p.Val1164Glyfs*7 | c.997A>T/c.3491T>G | 1 | PFIC2/ABCB11 |  |
| NN | p.Thr1210Pro | Homozygous c.3628A>C | 1 | PFIC2/ABCB11 |  |
| NN | Splicing+p.Lys461Glu | c.77-1G > A+c.1381A>G | 1 | PFIC2/ABCB11 |  |
| NN | p.Glu297Gly | Homozygous c.890A>G | 1 | PFIC2/ABCB11 |  |
| N | p.Trp493* | Homozygous c.1478G>A | 1 | PFIC2/ABCB11 |  |
| NN | p.Asp482Gly/p.Thr1210Pro | c.1445A>G/c.3628A>C | 1 | PFIC2/ABCB11 |  |
| N | splicing | Homozygous c.2178 + 1G > C | 2 | PFIC2/ABCB11 |  |
| NN | p.Asp482Gly+splicing | c.1445A>G+c.2178 + 1G > A | 1 | PFIC2/ABCB11 |  |
| NN | p.Asp482Gly | Homozygous c.1445A>G | 1 | PFIC2/ABCB11 |  |
| NN | p.Pro726Leu | Homozygous [c.2177C>T](javascript:window.location.hash%20=%20'00024077';%20return%20false) | 4 | PFIC3/ABCB4 |  |
| NN | Splicing+p.Gln1181Glu | c.286 + 1G > A+ c.3541C>G | 1 | PFIC3/ABCB4 |  |
| NN | p.Leu23Hisfs*16+p.Ser27Gly | -/c.79A>G | 1 | PFIC3/ABCB4 |  |

**Abbreviations:** n: number; N: null; NN: Non-null; PFIC: Progressive familial intrahepatic cholestasis; PFIC1: Progressive familial intrahepatic cholestasis type 1; PFIC2: Progressive familial intrahepatic cholestasis type 2; PFIC3: Progressive familial intrahepatic cholestasis type 3; PFIC4: Progressive familial intrahepatic cholestasis type 4.
